# Right ventricular pressure overload disturbs T-tubule maturation via MEF2D’s transcriptional regulation of BIN1

**DOI:** 10.1172/jci.insight.204032

**Published:** 2026-09-22

**Authors:** Yuqing Hu, Yiting Xue, Xudong Chen, Linghui Kong, Debao Li, Zheng Wang, Sixie Zheng, Siqi She, Hao Li, Sijuan Sun, Hao Chen, Lijun Chen, Peisen Ruan, Kai Wang, Lincai Ye

**Affiliations:** 1 Department of Cardiology,; 2 Department of Thoracic and Cardiovascular Surgery, Shanghai Children’s Medical Center, Shanghai Jiao Tong University School of Medicine, Shanghai, China.; 3 Department of Thoracic and Cardiovascular Surgery, Shanghai Children’s Hospital, Shanghai Jiao Tong University School of Medicine, Shanghai, China.; 4 Department of Ultrasound, Women and Children’s Health Care Hospital of Linyi, Linyi, Shandong, China.; 5 Department of Pediatric Surgery, Children’s Hospital of Fudan University, National Children’s Medical Center, Shanghai, China.; 6 Department of Pediatric Intensive Care Unit, Shanghai Children’s Medical Center, School of Medicine, Shanghai Jiao Tong University, Shanghai, China.; 7 Department of Pediatric Intensive Care Unit, The Affiliated Women and Children’s Hospital of Ningbo University, Ningbo, Zhejiang, China.; 8 Institute of Pediatric Translational Medicine and; 9 Shanghai Pediatric Congenital Heart Disease Institute, Shanghai Children’s Medical Center, Shanghai Jiao Tong University School of Medicine, Shanghai, China.

**Keywords:** Cardiology, Development, Cardiovascular disease

## Abstract

Right ventricular pressure overload (RVPO) is a critical pathophysiological feature of numerous pediatric cardiovascular diseases. Transverse tubules (T-tubules) form the foundation for efficient excitation-contraction coupling in mature cardiomyocytes. We hypothesized that RVPO impairs T-tubule maturation through the regulatory protein bridging integrator 1 (BIN1). In right ventricular samples from children with tetralogy of Fallot, characterized by RVPO, and in a neonatal rat RVPO model induced by pulmonary artery banding (PAB), T-tubule maturation was disrupted. RNA-seq revealed significant downregulation of T-tubule–associated genes, with *Bin1* among the most suppressed. *Bin1* overexpression restored T-tubule maturation in PAB rats. ATAC-seq showed reduced chromatin accessibility at *Bin1* loci; motif analysis identified *Mef2d* (myocyte enhancer factor 2D) as the top enriched transcription factor. *Mef2d* knockdown rescued *Bin1* expression and T-tubule maturation, and mutation of the *Mef2d* binding sites within the *Bin1* promoter abolished the inhibitory effect of *Mef2d* on *Bin1* promoter activity. This study delineates a phenomenon and a mechanism of cardiomyocyte maturation under pathological stress. The findings not only advance our understanding of this most pivotal event in postnatal cardiac development but also unveil a potential therapeutic direction for pediatric cardiovascular diseases associated with RVPO.

## Abstract 2

Right heart pressure overload impairs cardiomyocyte maturation via MEF2D–BIN1; restoring BIN1 rescues the defect.

## Graphical abstract

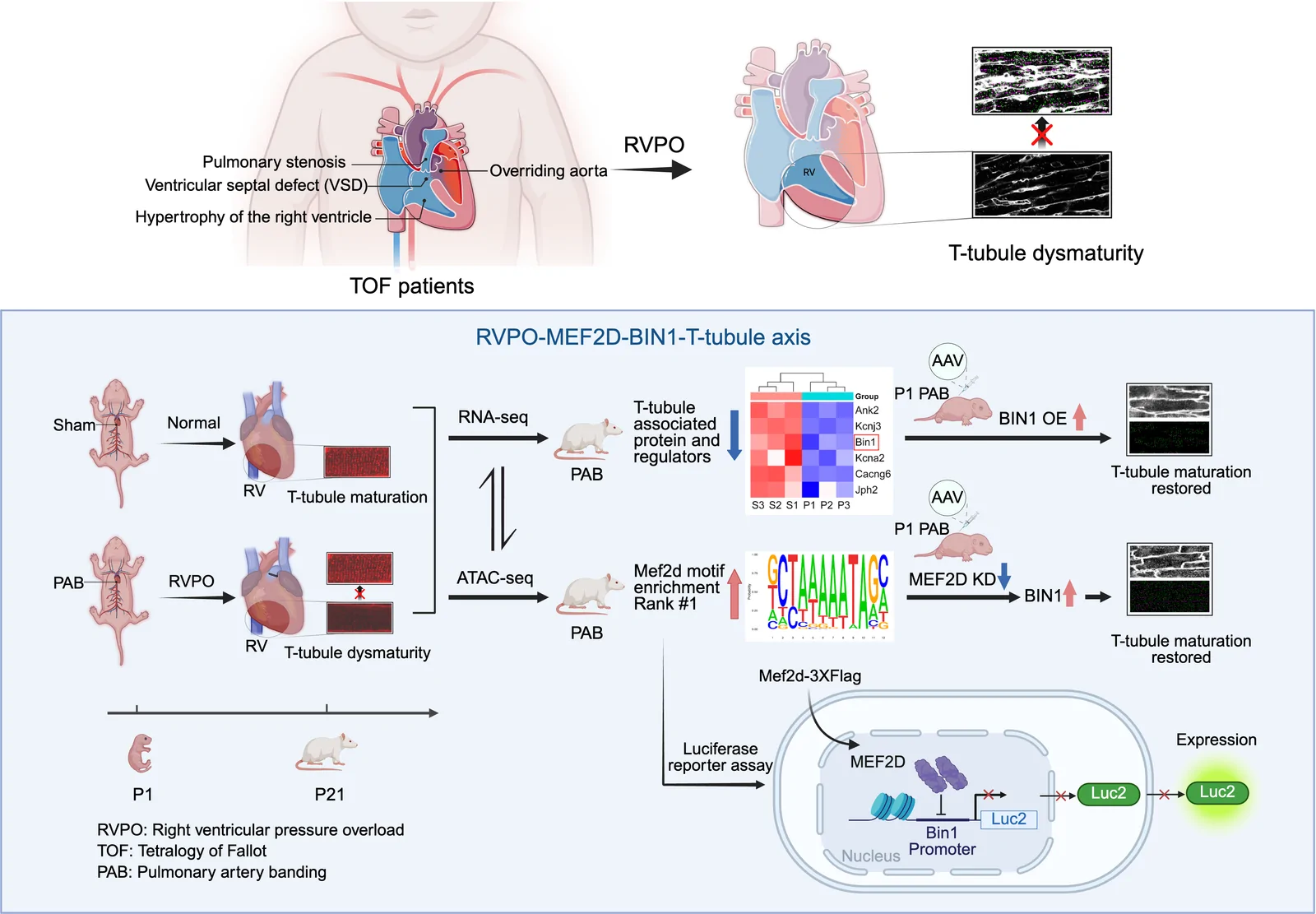

## Introduction

Right ventricular pressure overload (RVPO) is a critical pathophysiological feature of numerous pediatric cardiovascular diseases, including pulmonary arterial hypertension (PAH), tetralogy of Fallot (TOF), and pulmonary stenosis (PS) (1–4). It serves as a major determinant of survival and long-term quality of life in affected children (1–4). Despite its clinical importance, the influence of RVPO on postnatal right ventricular (RV) development remains poorly understood. Consequently, current pediatric treatment strategies are largely extrapolated from adult studies (5, 6). This approach is problematic, given the substantial molecular and electrophysiological differences between immature pediatric hearts and their adult counterparts (5, 6), often leading to limited efficacy in young patients (7, 8).

Cardiac immaturity — characterized by cardiomyocytes with underdeveloped transverse tubules (T-tubules) — is recognized as a potential contributor to functional impairments (6, 9). Notably, adult patients with TOF, even those who underwent optimal anatomical repair during childhood, remain at high risk for adverse outcomes including arrhythmias and sudden cardiac death (10). We hypothesize that RVPO in pediatric patients with TOF disrupts normal T-tubule maturation, leading to persistent structural and functional deficits in cardiomyocytes that may contribute to long-term vulnerability. Thus, elucidating how T-tubule maturation is compromised under RVPO may reveal potential therapeutic strategies to improve long-term outcomes.

T-tubules are specialized invaginations of the sarcolemma that form essential junctions with the junctional sarcoplasmic reticulum (jSR), enabling efficient excitation-contraction (E-C) coupling (11). During an action potential, membrane depolarization activates L-type calcium channels on T-tubules, which in turn trigger calcium release from ryanodine receptors (RyRs) on the jSR. This RyR-mediated calcium release generates a cytosolic calcium transient that initiates cardiomyocyte contraction (12).

Notably, T-tubules are absent at birth and develop postnatally (13). In rats, T-tubule biogenesis typically begins after the first postnatal week and reaches maturity around P21 (13). Recent studies have revised the traditional model of T-tubule formation; rather than arising solely from sarcolemmal invagination, emerging evidence indicates that T-tubules originate from intracellular membrane hubs (14). The process initiates with tubule budding from these hubs, followed by extension and interconnection to form a primitive network rich in longitudinal elements, which ultimately integrates with the sarcolemma (14).

The scaffolding protein bridging integrator 1 (BIN1) serves as a master regulator of this maturation process (14). BIN1 contains a conserved BAR domain that senses membrane curvature, a C-terminal SH3 domain that mediates protein-protein interactions, and a variable central region (15). Alternative splicing of its 20 exons generates multiple isoforms with distinct functions (15, 16). In human and rat hearts, the predominant isoforms are pBIN1 — which includes the BAR domain, a phosphoinositide-binding motif (PIBM) encoded by exon 11, and the SH3 domain — and cBIN1, which contains the BAR domain, SH3 domain, and exons 13 and 17 (14). Functionally, pBIN1 acts as the primary driver of membrane tubule budding and initial network formation, whereas cBIN1 sculpts microfolds on mature T-tubules to regulate ion channel localization and function (14). Importantly, pBIN1 is not detectable in mouse cardiomyocytes, suggesting fundamental differences in T-tubule formation mechanisms between mice and humans/rats (14). Based on this rationale, we selected rats as the experimental model for this study.

Despite its potential significance, whether and how RVPO affects T-tubule maturation remains entirely unexplored. This knowledge gap stems largely from the reliance of preclinical research on animal models (17, 18), coupled with a notable lack of neonatal RVPO models and the current inability of cardiac organoid systems to simulate RVPO (19, 20). Existing models are confined to adult or prepubertal animals (19, 20), in which T-tubules are already fully developed (13, 14), rendering them unsuitable for investigating developmental impairments.

In this study, we first identified impaired T-tubule maturation in pediatric patients with TOF. We then established a neonatal rat model of RVPO, as previously described (19, 21), which successfully recapitulated the T-tubule defects observed in human samples. Leveraging this model, we integrated RNA-seq, ATAC-seq, and genetic interventions — including knockdown, overexpression, and mutation — to uncover a potentially novel signaling axis, the RVPO/MEF2D/BIN1/T-tubule pathway, underlying the disruption of T-tubule maturation under RVPO.

## Results

### T-tubule maturation deficit in pediatric patients with RVPO.

**Figure 1:**
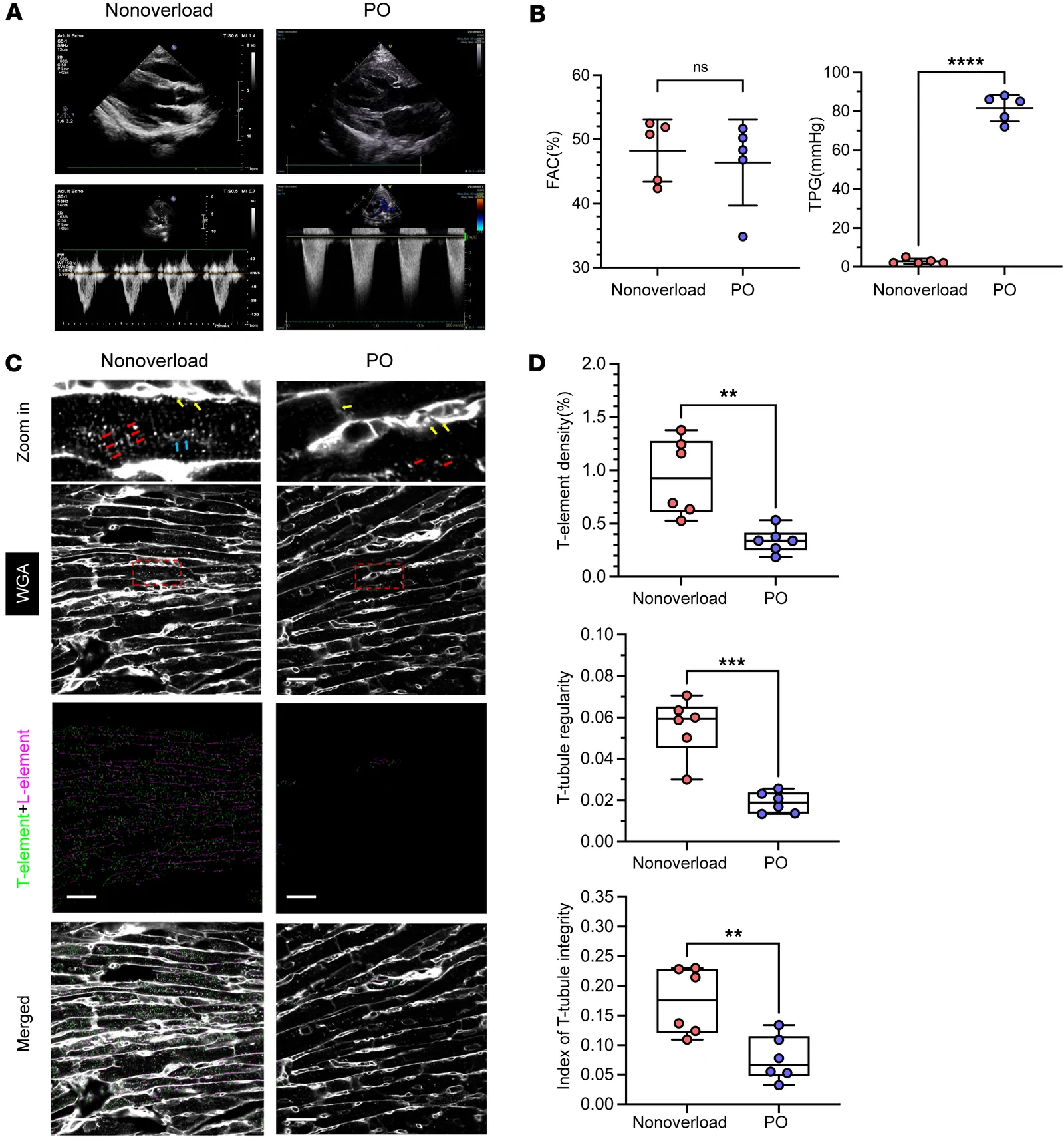
T-tubule maturation deficit in pediatric patients with RVPO. (**A**) Representative echocardiographic images from patients with ventricular septal defect (VSD, nonoverload group) and tetralogy of Fallot (TOF, pressure-overload group). (**B**) Quantification of Right Ventricular fractional area change (RV FAC) and transvalvular pressure gradient (TPG) measured by transthoracic echocardiography (*n* = 5 patients per group). (**C**) Representative images of T-tubules in the VSD and TOF groups. Scale bar: 25 μm. Zoomed-in view indicates red arrows that show T-elements, blue arrows indicate L-elements, and yellow lines denote the sarcolemma. (**D**) Quantification of T-element density, T-tubule regularity, and T-tubule integrity index (*n* = 6 rats per group, with 30 cardiomyocytes from 5 independent sections per rat). Data are presented as mean ± SD. *P* values were calculated by 2-tailed Student’s *t* test. **P < 0.01, ***P < 0.001, ****P < 0.0001.

To determine whether RVPO affects T-tubule maturation in humans, we analyzed myocardial samples from pediatric patients with or without RVPO, as assessed by transvalvular pressure gradient (TPG) (Table 1 and Figure 1, A and B). All patients exhibited preserved RV function, evaluated by RV Fractional area change (FAC) % due to the general unavailability of cardiac MRI data. In the pressure-overload (PO) group, T-element density, T-tubule regularity, and T-tubule integrity index were significantly reduced (0.961 ± 0.283, 0.019 ± 0.009, and 0.068 ± 0.036, respectively) compared with the nonoverload group (Figure 1, C and D). These findings indicate that RVPO may impede T-tubule maturation in humans. It should be noted that hypoxia was observed in some patients (Table 1), and its potential influence on T-tubule maturation requires further investigation.

### Establishment of a neonatal rat model of RVPO.

Given the central role of animal models in preclinical research (17, 18) and the prior lack of a neonatal RVPO model (19, 20), the effect of RVPO on postnatal RV development has remained unclear. We therefore established a neonatal RVPO model via pulmonary artery banding (PAB) on P1, as described previously (19, 21). At P7, pulmonary artery velocity-time integral (PA-VTI), peak velocity, and TPG were significantly elevated in PAB rats compared with sham controls (Supplemental Figure 1, A and B; supplemental material available online with this article; https://doi.org/10.1172/jci.insight.204032DS1). By P21, RV hypertrophy was evident, accompanied by ventricular septal shifting toward the left ventricle (LV) (Supplemental Figure 1, C and D). Although RV ejection fraction (RVEF) was generally preserved in PAB rats — consistent with observations in patients (3, 22) — a subset (~50%) exhibited reduced RVEF (Supplemental Figure 1, C and E). These results confirm the successful establishment of a neonatal RVPO model, providing a platform to investigate how RVPO remodels postnatal RV development.

### RVPO disrupts T-tubule maturation in neonatal rats.

**Figure 2:**
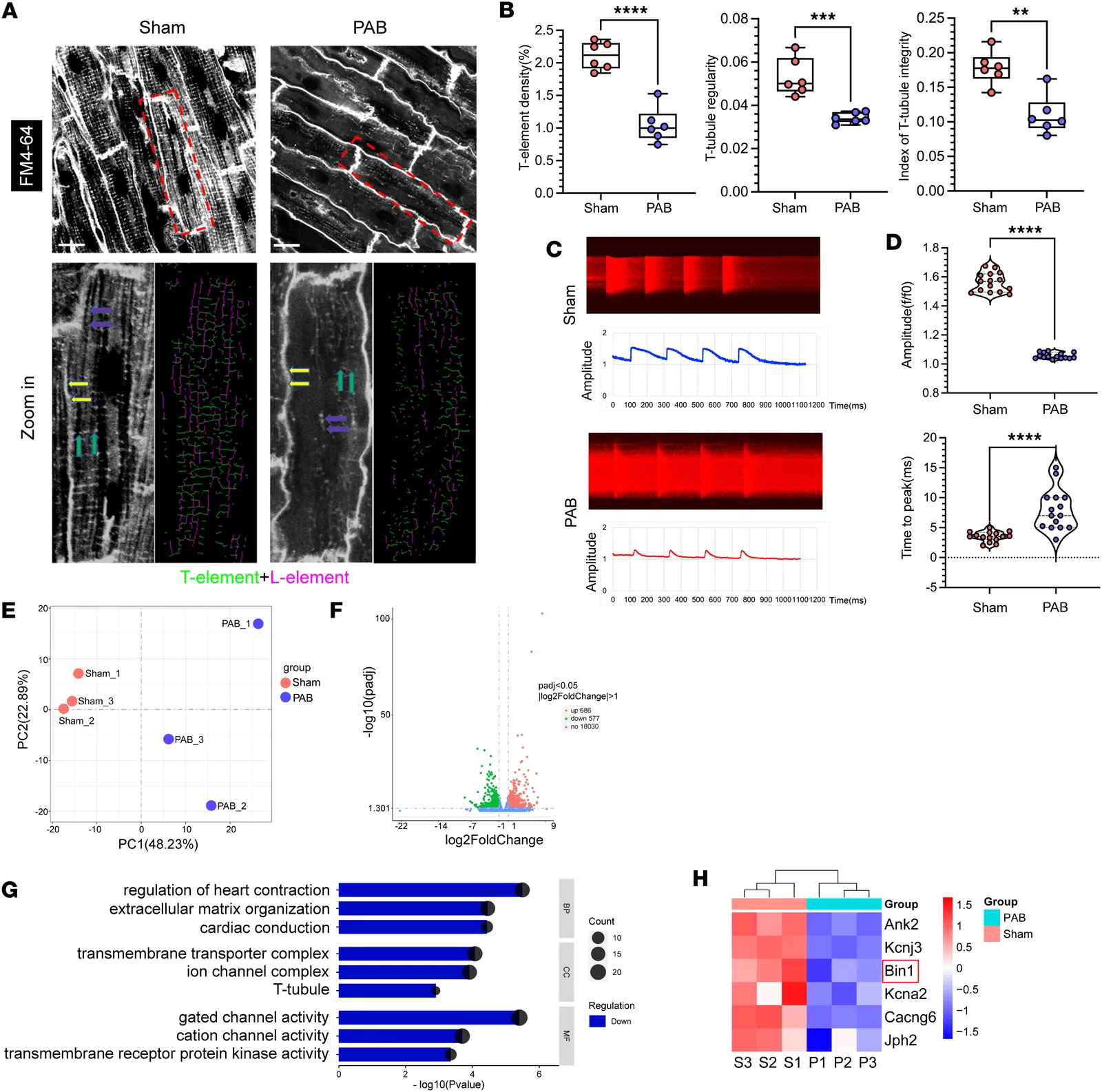
RVPO impairs T-tubule maturation in neonatal rats. (**A**) Representative T-tubule images from sham and PAB groups. Scale bar: 10 μm. Zoomed-in panels show green arrows that indicate T-elements, purple arrows that indicate L-elements, and yellow arrows that denote the sarcolemma. (**B**) Quantification of T-element density, T-tubule regularity, and T-tubule integrity index (*n* = 6 rats per group, with 30 cardiomyocytes from 5 independent sections per rat). (**C**) Upper panel: representative calcium transient traces from sham group cardiomyocytes. Lower panel: representative traces from PAB group cardiomyocytes. Note the elevated basal calcium signal intensity in PAB cardiomyocytes (~1.4) compared with sham (~1.0). (**D**) Quantification of calcium transient amplitude and time to peak (*n* = 15 cardiomyocytes per group; Mann-Whitney *U* test). (**E**) Principal component analysis of transcriptional profiles from sham and PAB samples. (**F**) Volcano plot of differentially expressed genes in PAB versus sham (adjusted *P* < 0.05, |log_2_FC| > 1). (**G**) Gene Ontology enrichment analysis of downregulated genes in PAB RVs. (**H**) Heatmap of downregulated genes in PAB-influenced RVs. Data are presented as mean ± SD. *P* values were calculated by 2-tailed Student’s *t* test. **P < 0.01, ***P < 0.001, ****P < 0.0001.

We next assessed whether the neonatal RVPO model recapitulated the T-tubule defects observed in patients. As shown in Figure 2, A and B, T-element density, T-tubule regularity, and T-tubule integrity index were all significantly lower in the PAB group than in sham controls, indicating impaired T-tubule formation in neonatal rats and mirroring the human phenotype.

Since T-tubules are essential for efficient calcium handling in cardiomyocytes (14), we evaluated intracellular calcium dynamics. The amplitude of calcium transients was significantly reduced, and the time to peak was prolonged in PAB cardiomyocytes relative to sham (Figure 2, C and D). Moreover, PAB cells exhibited elevated basal cytosolic calcium levels (Figure 2C), suggesting impaired sarcoplasmic reticulum calcium reuptake and further supporting T-tubule dysfunction. Together, these functional data indicate that RVPO disrupts T-tubule maturation.

To explore the underlying mechanisms, we performed RNA-seq analysis of RV cardiomyocytes. Principal component analysis (PCA) revealed distinct transcriptomic profiles between PAB and sham groups (Figure 2E). A total of 686 genes were upregulated and 577 were downregulated in PAB samples (adjusted *P* < 0.05, |log_2_FC| > 1; Figure 2F). Gene ontology (GO) enrichment analysis indicated that downregulated genes were associated with processes including heart contraction, cardiac conduction, T-tubule organization, ion channel complex assembly, gated channel activity, and cation channel activity (Figure 2G). Notably, *Bin1* and *Jph2* — 2 key T-tubule regulators (12, 14) — were among the downregulated genes in T-tubule–related GO terms (Figure 2H).

### Negative correlation between cardiomyocyte hypertrophy and T-tubule gene expression.

Because T-tubule remodeling is frequently accompanied by cardiomyocyte hypertrophy (Supplemental Figure 1, C and D), we performed single-nucleus RNA-seq on RV tissues from sham and PAB rats at P1, P7, P14, and P21 (Supplemental Figure 2, A–D). After quality control and clustering, we constructed cardiomyocyte-specific metacells using the high dimensional weighted gene coexpression network analysis (hdWGCNA) framework to reduce technical noise. Pearson correlation analysis on metacell expression profiles revealed a significant negative correlation between hypertrophy marker genes (*Nppa*, *Nppb*) and T-tubule–related genes (*Bin1*, *Jph2*) across all time points (Supplemental Figure 2, E–H). These results suggest that the development of hypertrophy and the impairment of T-tubule maturation are inversely linked during postnatal RVPO, although causality remains to be determined.

### RVPO alters chromatin accessibility of T-tubule–related genes.

**Figure 3:**
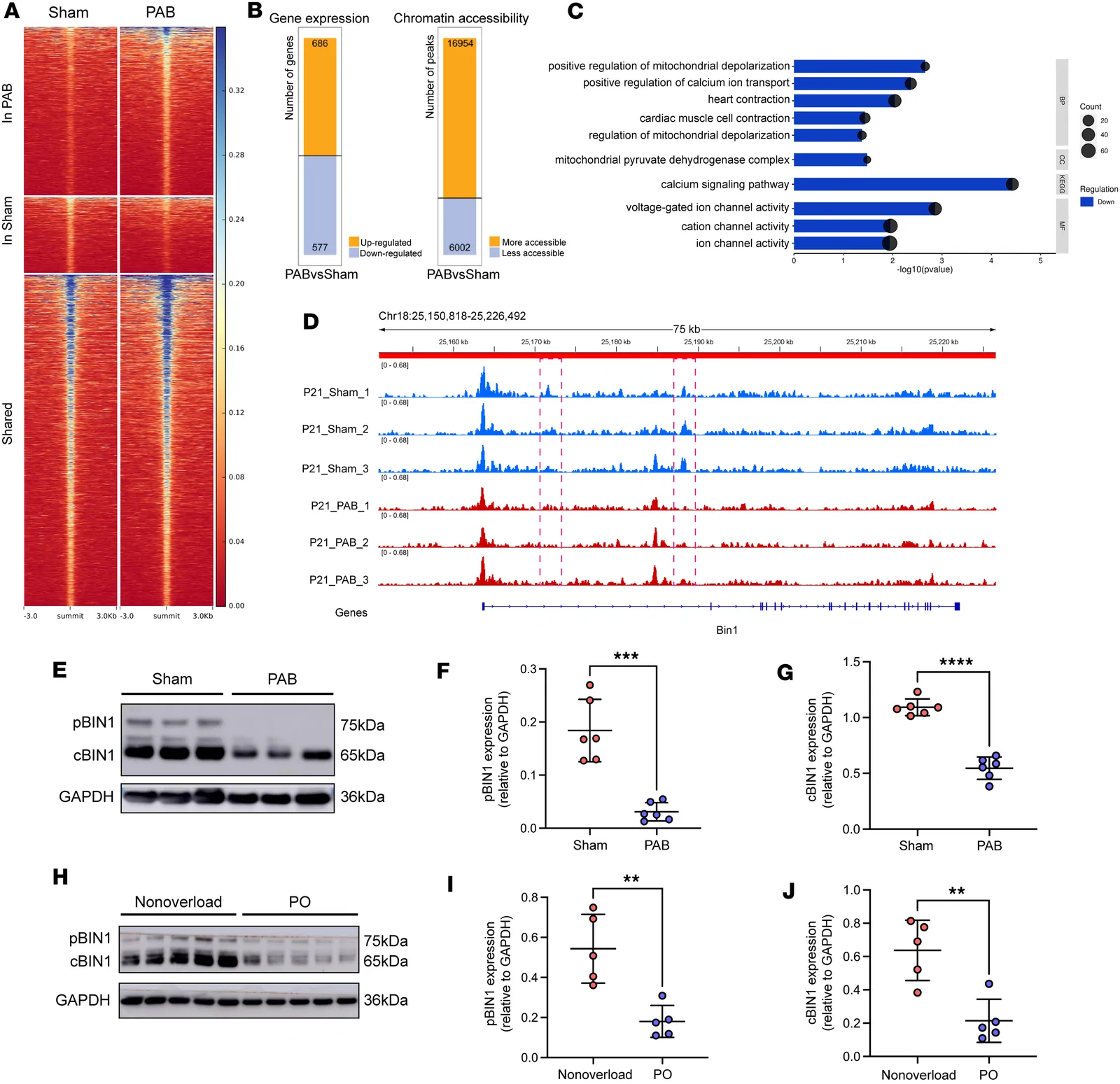
RVPO alters chromatin accessibility of T-tubule–associated genes. (**A**) Heatmap of shared and differentially accessible ATAC-seq peaks and associated genes. (**B**) Summary of differential gene expression and chromatin accessibility. “Up-regulated” and “Down-regulated” refer to RNA-seq results; “More accessible” and “Less accessible” refer to ATAC-seq results. (**C**) Enrichment analysis of genes with reduced chromatin accessibility in PAB RVs. (**D**) Chromatin accessibility profile around the transcription start site (TSS) of the *Bin1* gene. (**E**) Representative BIN1 Western blot in sham and PAB groups. (**F**) Quantification of pBIN1 protein expression (*n* = 6 rats per group). (**G**) Quantification of cBIN1 protein expression (*n* = 6 rats per group). (**H**) Representative BIN1 Western blot in VSD and TOF patient samples. (**I**) Quantification of pBIN1 expression in human samples (*n* = 5 patients per group). (**J**) Quantification of cBIN1 expression in human samples (*n* = 5 patients per group). Data are presented as mean ± SD. *P* values were calculated by 2-tailed Student’s *t* test. **P < 0.01, ***P < 0.001.

As chromatin accessibility governs transcriptional activity (23, 24), we performed ATAC-seq to assess its changes under RVPO. Approximately 57.57% of ATAC-seq peaks were shared between PAB and sham groups, while 29.28% (16,954 peaks) and 13.15% (6,002 peaks) were unique to PAB and sham groups, respectively (Figure 3, A and B). Genes with reduced accessibility in PAB (i.e., those corresponding to sham-only peaks) were enriched in biological processes such as heart contraction, mitochondrial depolarization, ion channel activity, and calcium signaling (Figure 3C) — findings consistent with RNA-seq findings and the impaired calcium handling observed in PAB cardiomyocytes (Figure 2, C–H).

Focusing on *Bin1* and *Jph2*, we observed reduced chromatin accessibility at the *Bin1* locus in PAB samples (Figure 3D), whereas JPH2 expression did not change in human RVPO samples but was downregulated in PAB rats (Supplemental Figure 3, A and B), and *Jph2* accessibility was increased (Supplemental Figure 3C). Given this divergent regulation, *Jph2* was excluded from subsequent mechanistic studies. Of note, this does not imply that *Jph2* is less important than *Bin1*; it may be regulated through distinct pathways.

We next validated these findings at the protein level. Both pBIN1 and cBIN1 were downregulated in PAB rat RVs (Figure 3, E–G). Importantly, a similar reduction was observed in human RVPO samples (Figure 3, H–J), suggesting that both BIN1 isoforms contribute to RVPO-induced deficits in T-tubule maturation.

### BIN1 overexpression rescues T-tubule maturation in neonatal RVPO rats.

**Figure 4:**
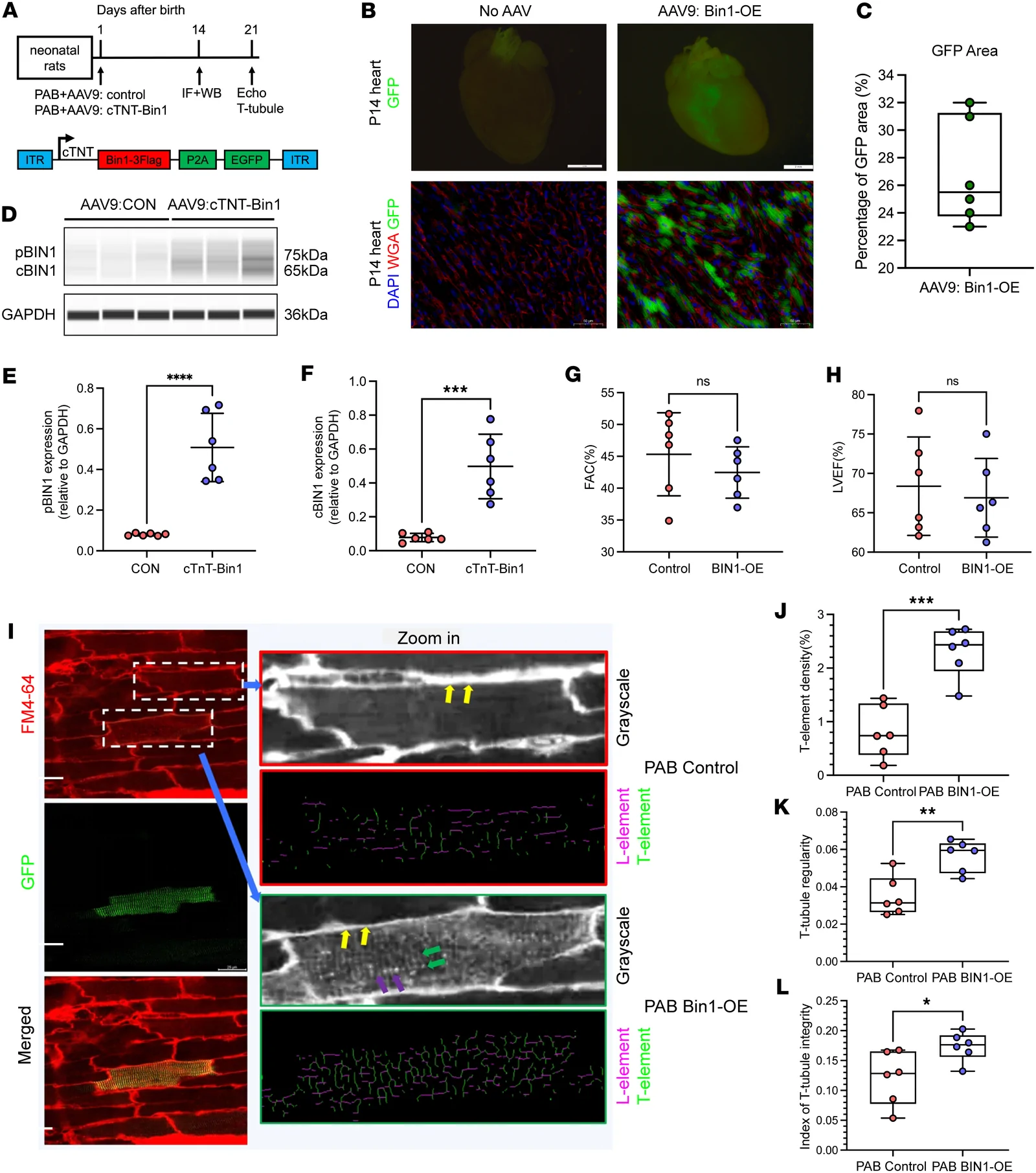
BIN1 overexpression rescues T-tubule maturation in neonatal RVPO rats. (**A**) Schematic of the BIN1 overexpression strategy. (**B** and **C**) AAV9 infection efficiency. (**D**) Representative BIN1 WES immunoblot from isolated RV cardiomyocytes. (**E**) Quantification of pBIN1 expression (*n* = 6 rats). (**F**) Quantification of cBIN1 expression (*n* = 6 rats). (**G**) Right ventricular fractional area change (RV FAC, %) of rats at P21. (**H**) Left ventricular ejection fraction (LVEF, %). (**I**) Representative T-tubule images from BIN1-overexpressing (BIN1-OE) and control cardiomyocytes in PAB RVs. Scale bar: 25 μm. Zoomed-in panels show green arrows that indicate T-elements, purple arrows that indicate L-elements, and yellow arrows that denote the sarcolemma. (**J–L**) Quantification of T-element density (**J**), T-tubule regularity (**K**), and T-tubule integrity index (**L**) (*n* = 6 rats per group, with 30 cardiomyocytes per rat). Data are presented as mean ± SD. *P* values were calculated by 2-tailed Student’s *t* test. *P < 0.05, **P < 0.01, ***P < 0.001, ****P < 0.0001.

Given the downregulation of both pBIN1 and cBIN1, we asked whether restoring BIN1 expression could ameliorate T-tubule defects. We overexpressed full-length BIN1 mRNA in neonatal RVPO rats using AAV9 vectors under the cTnT promoter (Figure 4A). An infection rate of 20%–30% was maintained to avoid altering global cardiac function (Figure 4, B and C) (25, 26). As expected, AAV-mediated *Bin1* delivery increased both pBIN1 and cBIN1 expression in cardiomyocytes (Figure 4, D–F), without affecting LV or RV function (Figure 4, G and H). This confirms that subsequent improvements in T-tubule integrity resulted directly from BIN1 upregulation rather than secondary hemodynamic changes.

To precisely evaluate BIN1’s role, we compared T-tubule architecture between BIN1-overexpressing (GFP^+^) cardiomyocytes and adjacent noninfected cells within the same RV microenvironment (Figure 4I). BIN1-overexpressing cells exhibited significantly higher T-element density, T-tubule regularity, and T-tubule integrity index (Figure 4, I–L), confirming that BIN1 restoration is sufficient to rescue T-tubule maturation in the setting of uncorrected RVPO. Furthermore, BIN1 overexpression also significantly reduced cardiomyocyte hypertrophy (Supplemental Figure 4)

### MEF2D mediates BIN1 downregulation in neonatal RVPO rats.

**Figure 5:**
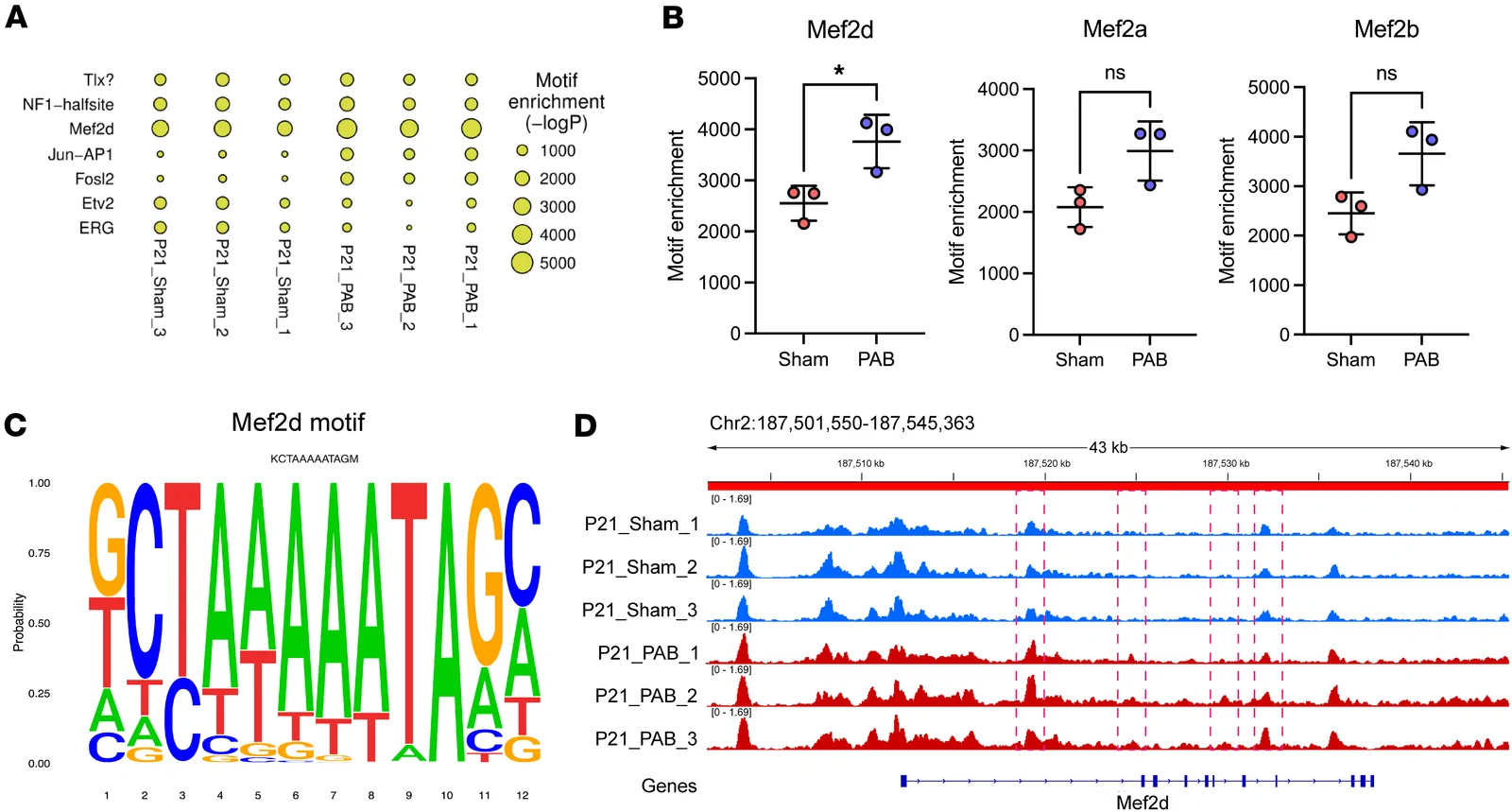
*Mef2d* motif is highly enriched in PAB rat RVs. (**A**) Dot plot showing enrichment of known transcription factor motifs. (**B**) Comparison of Mef2a, Mef2b, and *Mef2d* motif enrichment between sham and PAB RVs. (**C**) Sequence logo of the *Mef2d* motif. (**D**) Chromatin accessibility profile around the *Mef2d* TSS. Data are presented as mean ± SD. *P* values were calculated by 2-tailed Student’s *t* test. *P < 0.05.

To investigate the cause of *BIN1* downregulation, we analyzed transcription factor motif enrichment in the ATAC-seq data. The *Mef2d* motif was the most significantly enriched in PAB-specific accessible regions (Figure 5, A and B, and Table 2). Furthermore, chromatin accessibility at the *Mef2d* locus was higher in PAB than in sham rats (Figure 5, C and D), suggesting that *Mef2d* may contribute to *Bin1* repression. Moreover, MEF2D expression was also increased in human RVPO samples (Supplemental Figure 5), indicating that this regulatory mechanism is conserved across species.

**Figure 6:**
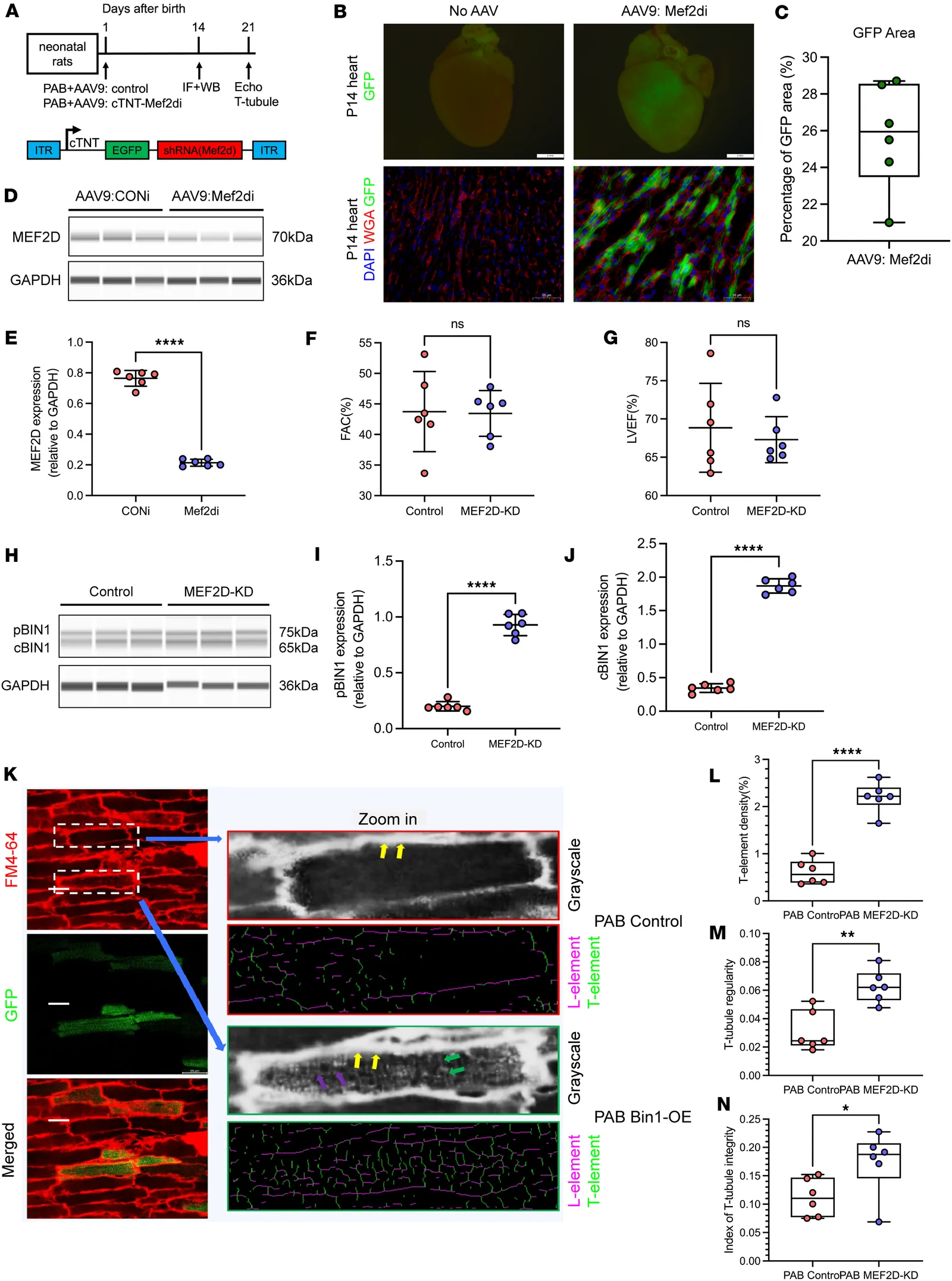
MEF2D knockdown rescues T-tubule maturation in neonatal RVPO rats. (**A**) Schematic of the MEF2D knockdown strategy. (**B** and **C**) AAV9 infection efficiency. (**D**) Representative MEF2D WES immunoblot from isolated RV cardiomyocytes. (**E**) Quantification of MEF2D expression (*n* = 6 rats). (**F**) Right ventricular fractional area change (RV FAC, %) of rats at P21. (**G**) Left ventricular ejection fraction (LVEF, %). (**H**) Representative BIN1 WES immunoblot from isolated cardiomyocytes. (**I**) Quantification of pBIN1 expression (*n* = 6 rats). (**J**) Quantification of cBIN1 expression (*n* = 6 rats). (**K**) Representative T-tubule images from MEF2D-knockdown (MEF2D-KD) and control cardiomyocytes in PAB RVs. Scale bar: 25 μm. Green arrows indicate T-elements, purple arrows indicate L-elements, and yellow arrows denote the sarcolemma. (**L**–**N**) Quantification of T-element density (**L**), T-tubule regularity (**M**), and T-tubule integrity index (**N**) (*n* = 6 rats per group, with 30 cardiomyocytes per rat). Data are presented as mean ± SD. *P* values were calculated by 2-tailed Student’s *t* test. *P < 0.05, **P < 0.01, ****P < 0.0001.

We therefore knocked down MEF2D in neonatal RVPO rats by AAV (Figure 6A). With the AAV infection rate at 20%–30% (Figure 6, B and C), we observed that knockdown of MEF2D in cardiomyocytes (Figure 6, D and E) did not significantly compromise left or RV function (Figure 6, F and G), thus excluding potential secondary effects from heart failure (25, 26). Importantly, it markedly upregulated the expression of BIN1 isoforms, including both pBIN1 and cBIN1(Figure 6, H–J). As a result, T-tubule maturation was restored, as indicated by increased T-element density, regularity, and index of TT integrity of T-tubules (Figure 6, K–N). Consistent with BIN1 overexpression, MEF2D knockdown also attenuated cardiomyocyte hypertrophy (Supplemental Figure 4).

**Figure 7:**
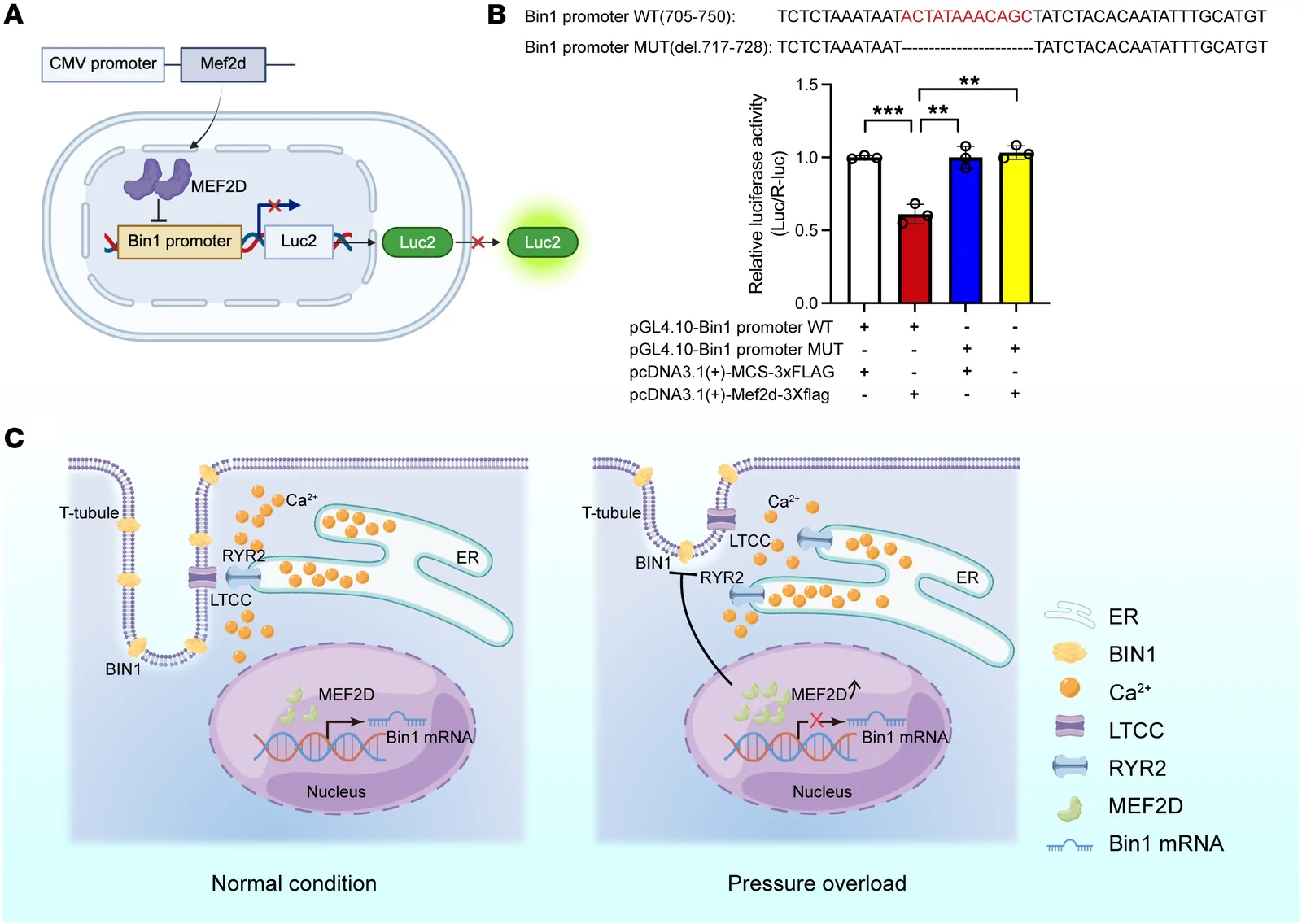
MEF2D inhibits *Bin1* promoter activity. (**A**) Schematic of the dual-luciferase reporter assay. (**B**) Luciferase activity assays evaluating MEF2D regulation of the *Bin1* promoter. Data were analyzed by Kruskal-Wallis test with post hoc Dunn’s test. **P < 0.01, ***P < 0.001. (**C**) Graphical summary of the RVPO/MEF2D/BIN1/T-tubule pathway identified in this study.

To determine whether MEF2D directly regulates *Bin1* transcription, we mutated the predicted *Mef2d*-binding sites in the *Bin1* promoter (Figure 7, A and B). Luciferase reporter assays showed that MEF2D significantly suppressed WT *Bin1* promoter activity, whereas mutation of the *Mef2d*-binding sites abolished this repression (Figure 7B). These results establish a direct regulatory pathway: RVPO → MEF2D → BIN1 → T-tubule maturation (Figure 7C).

## Discussion

This study elucidates a previously unrecognized molecular pathway through which RVPO disrupts a fundamental aspect of postnatal cardiac maturation: the development of the T-tubule network. We have identified and validated a RVPO/MEF2D/BIN1/T-tubule axis that operates in both pediatric patients with TOF and a corresponding neonatal rat model, providing a mechanistic explanation for the impaired cellular-level E-C coupling that may underpin long-term functional vulnerabilities in these hearts (3, 10).

The initial finding of disorganized and sparse T-tubules in the right ventricles of children with TOF directly links a common clinical condition (RVPO) to a core defect in cardiomyocyte ultrastructure. This observation is crucial because a mature T-tubule system is indispensable for synchronous calcium release and robust contraction (3, 10). The functional consequence of this structural deficit was confirmed in our neonatal RVPO model, which faithfully recapitulated the human phenotype. Cardiomyocytes from these rats exhibited compromised calcium handling, characterized by reduced amplitude and prolonged time-to-peak of calcium transients (Figure 2, C and D). These calcium kinetics are classic hallmarks of a deficient T-tubule network, where the loss of synchronous L-type calcium channel triggering leads to delayed and disorganized RyR activation (27, 28). The preservation of global RV function (as measured by FAC and LVEF) in the presence of such significant cellular-level dysfunction highlights a critical concept: the heart can compensate for impaired E-C coupling in its early stages, but this cellular deficit likely creates a substrate for arrhythmias and eventual contractile failure later in life, as seen in adult TOF survivors (1–2).

Our multiomics approach was pivotal in dissecting the mechanism upstream of this structural defect. The convergent evidence from RNA-seq and ATAC-seq was particularly powerful (1–2). RNA-seq revealed a broad downregulation of genes critical for E-C coupling and T-tubule integrity, while ATAC-seq demonstrated that this downregulation was associated with reduced chromatin accessibility in the regulatory regions of these same genes. This synergy suggests that RVPO instigates a transcriptional reprogramming that actively suppresses the cardiomyocyte maturation program. Among the downregulated genes, *Bin1* emerged as a prime candidate. The role of *Bin1* as a master regulator of membrane curvature and T-tubule formation is well established (3, 10), and its downregulation provides a direct molecular link to the observed structural deficits. Importantly, our study adds nuance by showing that both the tubule-forming engine pBIN1 and the microfold-sculpting cBIN1 are suppressed in RVPO, indicating a comprehensive disruption of the entire T-tubule maturation process. Consistently, we also observed reduced BIN1 expression in human TOF samples (Figure 3, H–J), confirming the clinical relevance of this finding.

The most significant mechanistic insight of this work is the identification of MEF2D as the transcriptional repressor responsible for BIN1 suppression. The enrichment of the *Mef2d* motif in the less-accessible chromatin regions of RVPO hearts, coupled with the increased openness of the *Mef2d* gene itself, painted a picture of MEF2D upregulation leading to the repression of its target genes. Our functional experiments confirmed this hypothesis. Knockdown of MEF2D in cardiomyocytes, while leaving global ventricular function intact, successfully restored BIN1 expression and, most importantly, rescued the T-tubule network. This finding is paradigm shifting. MEF2D, a transcription factor traditionally associated with promoting muscle differentiation and hypertrophy, is here revealed to act as a transcriptional repressor of a key maturation gene in the context of neonatal RVPO. The dual-luciferase assay provided the final piece of direct evidence, demonstrating that MEF2D binds to the *Bin1* promoter to inhibit its activity. This establishes a clear, direct line from the pathological stimulus (RVPO) to a transcriptional regulator (MEF2D), to an effector protein (BIN1), and finally to a cellular phenotype (impaired T-tubule maturation).

To assess whether this regulatory axis is conserved across species, we examined MEF2D expression in human RVPO samples. We collected an additional 4 samples per group (Table 3) and found that MEF2D expression was also significantly elevated in patients with TOF compared with controls. These results, together with the BIN1 changes observed in human samples, strongly suggest that the RVPO/MEF2D/BIN1/T-tubule pathway is evolutionarily conserved and relevant to human disease.

Another important aspect of RVPO pathophysiology is cardiomyocyte hypertrophy. Using single-nucleus RNA-seq of rat right ventricles from P1 to P21, we observed a significant negative correlation between the expression of hypertrophy marker genes (*Nppa*, *Nppb*) and T-tubule–related genes (*Bin1*, *Jph2*). This finding suggests that hypertrophy and T-tubule remodeling are inversely related during postnatal development under pressure overload. Whether hypertrophy directly impairs T-tubule maturation or vice versa remains to be determined; nevertheless, our data highlight a potential reciprocal interaction that warrants further investigation.

The therapeutic implications of this axis are substantial. Our rescue of T-tubule structure through BIN1 overexpression, even in the persistent presence of RVPO, is a proof of concept that targeting this pathway can reverse cellular-level pathology. This suggests that strategies aimed at enhancing BIN1 expression or function — or inhibiting the repressive action of MEF2D — could be developed to promote myocardial maturation and resilience in children with RVPO, potentially improving their long-term outcomes independently of surgical correction of the overload itself.

Our preclinical experiments included both male and female neonatal rats with approximately equal sex distribution, and we observed no obvious sex-dependent differences in the RVPO-induced T-tubule defects or BIN1 downregulation in post hoc analyses. However, because all human tissue samples were derived from male patients, the generalizability of these findings to female pediatric patients, and the potential for sex-specific modulation of the MEF2D/BIN1 axis, warrant further investigation in larger, sex-balanced clinical cohorts.

Several important considerations arise from our work. First, the focus of this study was on the early, neonatal response to RVPO. It will be critical to investigate whether this MEF2D-mediated repression is sustained into adulthood and contributes to the progression of heart failure. Second, while we established the role of this axis in cardiomyocytes, the potential crosstalk with other cardiac cell types, such as fibroblasts and endothelial cells, remains an open question. Paracrine signals originating from these cells in the PO ventricle could further modulate the MEF2D/BIN1 axis in cardiomyocytes. Third, the upstream signal that triggers MEF2D upregulation in response to RVPO — whether mechanical stress, neurohormonal activation, or metabolic changes — is a vital area for future investigation. Fourth, we acknowledge that our identification of pBIN1 and cBIN1 isoforms was based on molecular weight differences reported in the literature (Supplemental Figure 6) (14) and the antibody manufacturer’s datasheet, as no commercially available antibodies specifically recognizing individual exons of BIN1 are currently available. Future studies using exon-specific antibodies or RNA probes will be valuable to further dissect the distinct functions of each isoform. Fifth, the absence of dedicated electrophysiological studies — such as electrocardiography (ECG) and programmed electrical stimulation — precludes a definitive assessment of arrhythmia susceptibility in this model. Therefore, while our calcium transient data suggest functional impairment, we refrain from making direct claims regarding arrhythmogenesis. Finally, although our in vivo data strongly support the role of MEF2D in T-tubule homeostasis, future in vitro studies using siRNA knockdown in adult cardiomyocytes or induced pluripotent stem cell–derived cardiomyocytes could provide complementary evidence and help elucidate the direct maintenance functions of MEF2D.

In conclusion, our study unveils a pathological mechanism wherein RVPO drives the upregulation of the transcription factor MEF2D, which in turn represses the expression of the key T-tubule sculpting protein BIN1, leading to a failure in T-tubule maturation and impaired calcium-mediated E-C coupling. The identification of this RVPO/MEF2D/BIN1/T-tubule axis not only deepens our understanding of the pathophysiology of pediatric heart diseases but also illuminates potential therapeutic avenues aimed at promoting proper myocardial maturation to safeguard the long-term health of these vulnerable young hearts (3, 10).

## Methods

### Sex as a biological variable.

All of the human samples are from male patients (Table 1 and Table 3). All rodent experiments were performed on both male and female rats. We observed no obvious sex-dependent differences in the RVPO-induced T-tubule defects or BIN1 downregulation in post hoc analyses. As such, sex was not evaluated as a biological variable in the animal experiments.

Human myocardial samples were obtained from male patients only, as tissue availability from female patients was limited during the study period. In the neonatal rat experiments, both male and female pups were included with approximately equal sex distribution (50% male, 50% female). No sex-dependent differences were observed in the RVPO-induced T-tubule defects or BIN1 downregulation in post hoc analyses. However, because all human samples were from male patients, the generalizability of these findings to female patients warrants further investigation.

### Human tissue samples.

RV outflow tract myocardial specimens were obtained from 5 pediatric patients with TOF (PO group) or ventricular septal defect (VSD, control group) at Shanghai Children’s Medical Center between February 2020 and May 2021. Each sample was preserved in liquid nitrogen and divided into 3 portions for WGA staining and Western immunoblotting analyses. All procedures involving human tissues complied with the principles of the Declaration of Helsinki and were approved by the institutional ethics committee. Written informed consent was obtained from the parents or guardians of all participants prior to inclusion in the study.

### WGA staining.

T-tubules in human heart samples were visualized using wheat germ agglutinin (WGA, Invitrogen, W32464) staining. Briefly, slides were washed 3 times with PBS and incubated with WGA for 30 minutes at room temperature. Images were acquired using a confocal microscope equipped with a 60×/1.3 silicon-oil objective and analyzed with AutoTT software as previously described (29). AutoTT performs automated quantification of the T-tubule network by preprocessing and segmenting raw confocal images to generate a binary mask, which is subsequently skeletonized to extract key parameters such as T-tubule density (including longitudinal and transverse components) and the Regularity Index, derived from the power spectrum of line scans. The output includes both numerical data and processed images for verification.

### Animals and surgical procedures.

Pregnant Sprague-Dawley rats were obtained from Xipu’er-bikai Experimental Animal Co., Ltd. Neonatal pups of both sexes, with approximately equal sex distribution, underwent PAB on P1, as described in our previous publications (19, 21). Briefly, pups were preanesthetized with 5% isoflurane, placed on an ice bed, and positioned supine. A horizontal thoracotomy was performed to expose the pulmonary artery (PA). An 11-0 nylon suture was passed beneath the PA alongside a 27-gauge needle (0.41 mm diameter). The suture was tightened around both the PA and the needle, after which the needle was removed to create a fixed constriction in the PA lumen. The thoracic wall was closed, and local lidocaine was applied for analgesia before pups were returned to their mother.

### Echocardiography.

Rats were anesthetized with 2.5% isoflurane at P7 and P21 and allowed to breathe spontaneously via a nasal cone (maintained at 1.5%–2.0% isoflurane in oxygen). Echocardiographic examinations were performed using a Vevo 2100 imaging system (Visual Sonics). The TPG was measured from a long-axis view of the PA using continuous-wave Doppler. Fractional area change (FAC) was determined by tracing the endocardial border in the apical 4-chamber view at end-diastole and end-systole.

### Histology.

Hearts were harvested after cervical dislocation, fixed in 4% paraformaldehyde overnight at room temperature, dehydrated in a graded ethanol series, embedded in paraffin, and sectioned at 6 μm thickness. H&E staining was performed using a commercial kit (C0105M, Beyotime Biotech) according to the manufacturer’s instructions.

### Cardiac MRI.

RV function was assessed by cardiac MRI at P21 using a 7-T Bruker Biospec 70/20 cm scanner (Ettlingen) equipped with a 300 mT/m gradient and IntraGate self-gating. End-systolic images were acquired in a multislice short-axis view covering the entire RV. Six contiguous axial slices were obtained, and the midpoint of the RV on the fourth slice (from the apex) was used to measure RV free-wall thickness (RVWT) and RV end-diastolic diameter. RV volume was calculated from all contiguous slices, and RVEF was derived as follows: RVEF = ([RVEDV – RVESV]/RVEDV) × 100%.

### In situ T-tubule imaging and analysis.

Hearts from P21 rats were rapidly excised following cervical dislocation and then perfused via the aorta with Ca^2+^-free Tyrode’s solution for 3 minutes using a Langendorff system. This was followed by perfusion with FM4-64 (Invitrogen, F34653) for 15 minutes. T-tubule images were captured using a confocal microscope with a 60×/1.3 silicon-oil objective and analyzed with AutoTT as previously described (29). For each animal, T-tubule quantification was performed on 30 randomly selected cardiomyocytes per rat. The mean value of these 30 cells was used to represent a single biological replicate for that animal.

### Cardiomyocyte isolation and calcium imaging.

Cardiomyocytes were isolated using a Langendorff perfusion system as previously reported (30). Only the right atrium (RA) was removed after perfusion, and RA-derived cardiomyocytes were used for calcium imaging. Prior to calcium and contractility analyses, cells were gradually reexposed to calcium using a series of 10 mL perfusion buffers containing 100 nmol/L, 400 nmol/L, 900 nmol/L, and 1.2 μmol/L CaCl_2_. Cardiomyocytes were allowed to settle by gravity for 10 minutes at room temperature at each step before transfer to the next higher calcium concentration. Cells were loaded with 5 μmol/L Rhod-4-AM (AAT Bioquest, 21121) for 20 minutes, washed with normal Tyrode’s solution (in mmol/L: 140 NaCl, 4 KCl, 1 MgCl_2_, 1.8 CaCl_2_, 10 glucose, 5 HEPES, pH 7.4 with NaOH), and settled in a laminin-coated glass-bottom chamber at 30°C for 10 minutes. Cells were electrically stimulated at 1 Hz to achieve steady-state conditions. Calcium signals were acquired via confocal line scanning along the long axis of the cell (avoiding the nucleus) using a 63× objective. Signals were quantified manually using Fiji software (v2.9.0).

### RNA extraction.

Rats were anesthetized with 1.5% isoflurane at P21 before sacrifice and RV collection. RNA was extracted using the PureLink RNA Micro Scale Kit (Thermo Fisher Scientific). RNA integrity was assessed with the RNA Nano 6000 Assay Kit on a Bioanalyzer 2100 system (Agilent Technologies). qPCR was performed using SYBR Green Power Premix kits (TaKaRa Bio), according to the manufacturer’s instructions, with primers supplied by Generay Biotech Co. Ltd.

### Library preparation.

Sequencing libraries were constructed using the NEBNext Ultra RNA Library Prep Kit for Illumina (NEB). Library fragments were purified with the AMPure XP system (Beckman Coulter), and quality was assessed on an Agilent Bioanalyzer 2100.

### Clustering, sequencing, and mapping.

Indexed samples were clustered on a cBot Cluster Generation System using the TruSeq PE Cluster Kit v3-cBot-HS (Illumina). Sequencing was performed on an Illumina NovaSeq platform to generate 150 bp paired-end reads. Raw reads in FASTQ format were processed using in-house Perl scripts to obtain clean reads. Downstream analyses were based on clean, high-quality data. The reference genome index was built using Hisat2 v2.0.5, and paired-end clean reads were aligned accordingly. Read counts per gene were obtained with featureCounts v1.5.0-p3, and FPKM values were calculated based on gene length and mapped read counts.

### Differential gene expression analysis.

Differential expression analysis was conducted using the DESeq2 R package (v1.16.1). Genes with an adjusted *P* < 0.05 were considered differentially expressed.

### GO enrichment analysis.

GO enrichment analysis of downregulated genes was performed using the clusterProfiler R package. GO terms with a corrected *P* < 0.05 were deemed significantly enriched.

### ATAC-seq sample preparation.

Frozen RV tissue was minced, resuspended in homogenization buffer, and ground into a homogeneous solution before filtration through a cell strainer. Cell pellets were collected by centrifugation (500*g*, 4 °C, 5 min). Nuclei were isolated via iodixanol density gradient centrifugation (3,000*g*, 4°C, 20 min), and 50,000 nuclei were used for the ATAC-seq.

### ATAC-seq library preparation and sequencing.

ATAC-seq was performed as previously described (31). Briefly, nuclei were extracted and resuspended in a Tn5 transposase reaction mix, followed by incubation at 37°C for 30 minutes. After transposition, equimolar Adapter 1 and Adapter 2 were added, and libraries were amplified by PCR. Amplified libraries were purified with AMPure beads, and quality was assessed using a Qubit fluorometer. Clustering and sequencing were carried out on a cBot Cluster Generation System and an Illumina HiSeq platform, respectively, to generate 150 bp paired-end reads.

### ATAC-seq data analysis.

Adapter sequences were trimmed from reads using Skewer (v0.2.2). Clean reads were aligned to the reference genome with BWA using standard parameters. High-quality (MAPQ ≥ 13), nonmitochondrial, properly paired reads longer than 18 nt were retained. Peak calling was performed with MACS2 using the command macs2 callpeak --no-model --keep-dup all --call-summits. Simulations of peaks per input read used aligned and deduplicated BAM files without additional filtering.

### Western blotting.

Fresh RV tissues or isolated cardiomyocytes were lysed in RIPA buffer (Beyotime, P0013B). Lysates were centrifuged at 12,000 rpm 13,700*g* for 10 minutes, and supernatants were boiled in 4× Laemmli buffer after protein concentration determination with a BCA kit (Beyotime, P0012S). Proteins were separated by SDS-PAGE, transferred to 0.45 μm PVDF membranes (MilliporeSigma), and probed with primary antibodies against BIN1 (Abcam, ab185950, 1:1,000), MEF2D (Novus Bio, NBP1-85788, 1:500), GAPDH (Sigma, G9545, 1:1,000), and JPH2 (Santa Cruz, sc-377086, 1:200), followed by incubation with HRP-conjugated secondary antibodies (Cell Signaling, 7074, 1:2,000). Signals were detected with enhanced chemiluminescence substrate (Immobilon Western, Millipore, WBKLS) on an Amersham Imager 600. Quantification was performed using ImageJ (NIH).

For samples with limited cell numbers from enzymatic digestion and flow cytometric sorting, protein expression was analyzed using the WES system (ProteinSimple). Samples were prepared per the manufacturer’s instructions using the separation (SM-W004) and detection (DM-001) kits. Primary antibodies against MEF2D, BIN1, JPH2, and GAPDH were used. Automated protein detection and data analysis were conducted with Compass for Simple Western software (v5.0.1).

### BIN1 overexpression.

To achieve cardiomyocyte-specific BIN1 overexpression, neonatal rats received a s.c. injection of AAV9 vectors carrying full-length *Bin1* mRNA under the control of the cardiac troponin T (cTnT) promoter (AAV9:cTNT-*Bin1*, Obio Biotechnology). Control animals received AAV9 expressing EGFP only (AAV9:CON). A total of 1 × 10^11^ viral particles were administered 1 hour before PAB surgery at P1. Infection efficiency was assessed by fluorescence imaging at 2 weeks after injection or by WES at endpoint. An infection rate of 20%–30% was maintained to avoid global cardiac functional compromise while allowing observation of single-cell gene effects (23).

### MEF2D knockdown.

For cardiomyocyte-specific MEF2D knockdown, rats were injected s.c. with AAV9 vectors expressing shRNA against *Mef2d* (AAV9:cTNT-Mef2di) or a scrambled control (AAV9:CONi). The target sequence for *Mef2d* shRNA was 5′-CACATCAGCATCAAGTCAGAA-3′. A total of 1 × 10^11^ viral particles were injected 1 hour before PAB surgery. The cTnT promoter was selected to ensure cardiomyocyte-specific knockdown, avoiding potential off-target effects in nonmyocyte cells (31). This approach was also chosen for its translational potential over transgenic *Mef2d*^fl/fl^ models (32).

### Dual-luciferase reporter assay.

HEK293T cells were seeded in 24-well plates and transfected with 1 μg of either truncated or mutated *Bin1* promoter luciferase reporters, along with 0.5 μg of empty vector or *Mef2d* expression plasmid. After 48 hours, luciferase activity was measured using the Dual-Luciferase Reporter Assay System (Promega) on a Varioskan Flash instrument (Thermo Fisher Scientific).

### Single-nucleus RNA-Seq sample preparation and sequencing.

RV tissues were collected from neonatal rats in the sham and PAB groups at 4 time points: P1, P7, P14, and P21 (Supplemental Figure 1A). Fresh frozen tissues were minced and homogenized in ice-cold lysis buffer. Nuclei were isolated using density gradient centrifugation (iodixanol-based) (3,000*g*, 4 °C, 20 min) and stained with DAPI. High-quality nuclei were sorted by FACS (BD FACSMelody) and immediately processed for library preparation using the Chromium Next GEM Single Cell 3’ Kit (v3.1, 10x Genomics) according to the manufacturer’s protocol. Sequencing was performed on an Illumina NovaSeq 6000 platform with a target depth of ≥ 50,000 reads per nucleus.

### Single-nucleus RNA-Seq data processing.

Raw sequencing data were demultiplexed and aligned to the rat reference genome (rn6) using Cell Ranger (v7.1.0). Downstream analyses were conducted using the Seurat R package (v5.3.0) (33). Low-quality nuclei were filtered out based on the following criteria: number of detected genes between 500 and 8,000, total UMI counts between 1,000 and 25,000, mitochondrial gene proportion < 20%, erythrocyte gene signature < 1%, and log_10_(Genes per UMI) > 0.7. Doublets were identified and removed using scDblFinder (v1.20.2) with default parameters (34). Data from different samples were integrated using the Harmony algorithm (v1.2.4) to correct for batch effects (35). Cell cycle scores were calculated, and both cell cycle effects and ribosomal gene percentages were regressed out during scaling. Dimensionality reduction and unsupervised clustering were performed using UMAP and t-SNE. Cell types were annotated by combining automated annotation with SingleR (36) against the CellMarker 2.0 database (37), followed by manual curation based on known cardiac cell markers (38, 39).

### Metacell construction and correlation analysis between hypertrophy and T-tubule gene expression.

To reduce technical noise inherent in single-nucleus data and to robustly assess coexpression relationships, we constructed cardiomyocyte-specific metacells using the hdWGCNA framework (40). Cardiomyocyte subpopulations were isolated based on the expression of canonical markers (Tnnt2, Myl2, Myh6). Metacells were generated using the MetacellsByGroups function with a k-nearest neighbor (KNN) approach (k = 25) within the Harmony-integrated reduction space, generating metacells separately for each experimental group (sham versus PAB) and each time point to preserve biological heterogeneity. The resulting metacell object was normalized, scaled, and subjected to PCA, with residual batch effects further corrected by Harmony.

To explore the relationship between cardiomyocyte hypertrophy and T-tubule maturation, we performed Pearson correlation analysis on metacell expression profiles. Hypertrophy marker genes (*Nppa*, *Nppb*) and T-tubule–related genes (*Bin1*, *Jph2*) were selected based on our RNA-seq and ATAC-seq results. Correlation coefficients (R) and *P* values were calculated using linear regression. Coexpression patterns were visualized using dual-feature UMAP plots. This metacell-based approach provided a robust statistical framework to identify coordinated expression signatures that might otherwise be masked by dropout events in raw single-cell measurements.

### Statistics.

Data are presented as mean ± SD. Statistical analyses were performed using SAS v11.2 (SAS Institute Inc.). For normally distributed data, two-tailed Student’s *t* test was used to evaluate differences between two groups, and one-way analysis of variance (ANOVA) followed by post hoc Dunn’s test was applied for multiple-group comparisons. For non-normally distributed data, Mann-Whitney *U* test was performed for two-group analysis, and Kruskal-Wallis test with post hoc Dunn’s test was used for multiple-group statistical comparison. A *P* value < 0.05 was considered statistically significant.

### Study approval.

All the procedures in this study conformed to the principles outlined in the Declaration of Helsinki and were approved by the Animal Welfare and Human Studies Committee at Shanghai Children’s Medical Center (IRB approval no. SCMCIRB-Y2020094 and no. SCMCIRB-K2022146-1).

### Data availability.

All data generated or analyzed in the article are included in the Supporting Data Values file. The RNA-seq and ATAC-seq datasets have been deposited in the Gene Expression Omnibus (GEO) database under accession no. GSE210180.

## Author contributions

YH designed the experiments, conducted experiments, analyzed data, and wrote the original draft of the manuscript. YX assisted with the experiments, discussed data, and edited the manuscript. XC and LK assisted with the experiments and discussed data. DL, LK, and LC assisted with the experiments. ZW discussed data and edited the manuscript. SZ, S She, HL, S Sun, and HC edited the manuscript. PR and KW design the experiments. LY conceptualized and designed the experiments, interpreted data, and wrote and edited the manuscript. YH, YX, XC, and LK contributed equally to this work. The order of co–first authors was determined based on their relative contributions to the study. All authors revised the manuscript and approved the final version of the manuscript.

## Conflict of interest

YH is applying a patent for treating pediatric patient with RVPO (Applying No. CN2025103492542).

## Funding support

- National Key Special Science and Technology Project (2025ZD0552203).
- National Natural Science Foundation of China (nos. 82400363, 82370832, and 82270314).
- Fundamental Research Funds for the Central Universities (project no. YG2024QNA47).
- Ningbo Top Medical and Health Research Program (no. 2022020405).
- Science and Technology Development Fund of Pudong New Area (PKJ2024-Y13 and PKJ2024-Y15).
- Medical and Health Technology Plan Projects of Zhejiang (2024KY1568).
- Ningbo Natural Science Foundation project (2024J334, 2024J294).
- Ningbo Medical and Health Brand Discipline (PPXK2024-06).
- National Clinical Key Specialty Construction Project(10000015Z155080000004).
- Shanghai Research Center for Pediatric Cardiovascular Diseases (2023ZZ02024).
- Shanghai Key Laboratory of Clinical Molecular Diagnostics for Pediatrics (20dz2260900).
- Innovative research team of high-level local universities in Shanghai.
- Shanghai Research Center for Pediatric Cardiovascular Diseases (2023ZZ02024).
- The Project of Guizhou Provincial Science and Technology Agency (KJLYRC-[2026]045).
- Science and Technology Project of Guizhou Province (KXJZ[2024]035).
- Science and Technology Project of Guizhou Province (LC[2026]281).

## Supplementary Material

Supplemental data

Unedited blot and gel images

Supporting data values

## Figures and Tables

**Table 1:**
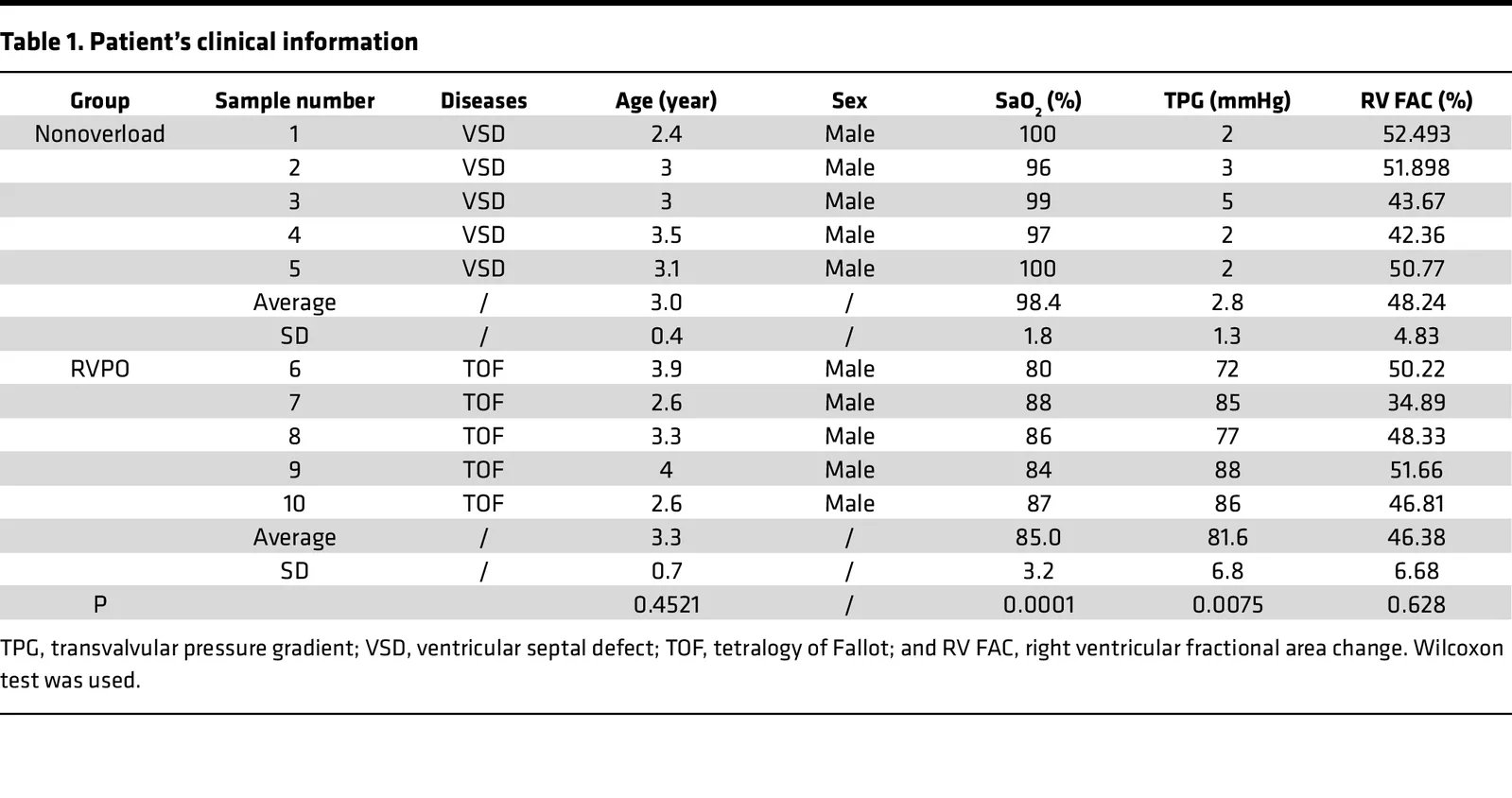
Patient’s clinical information

**Table 2:**
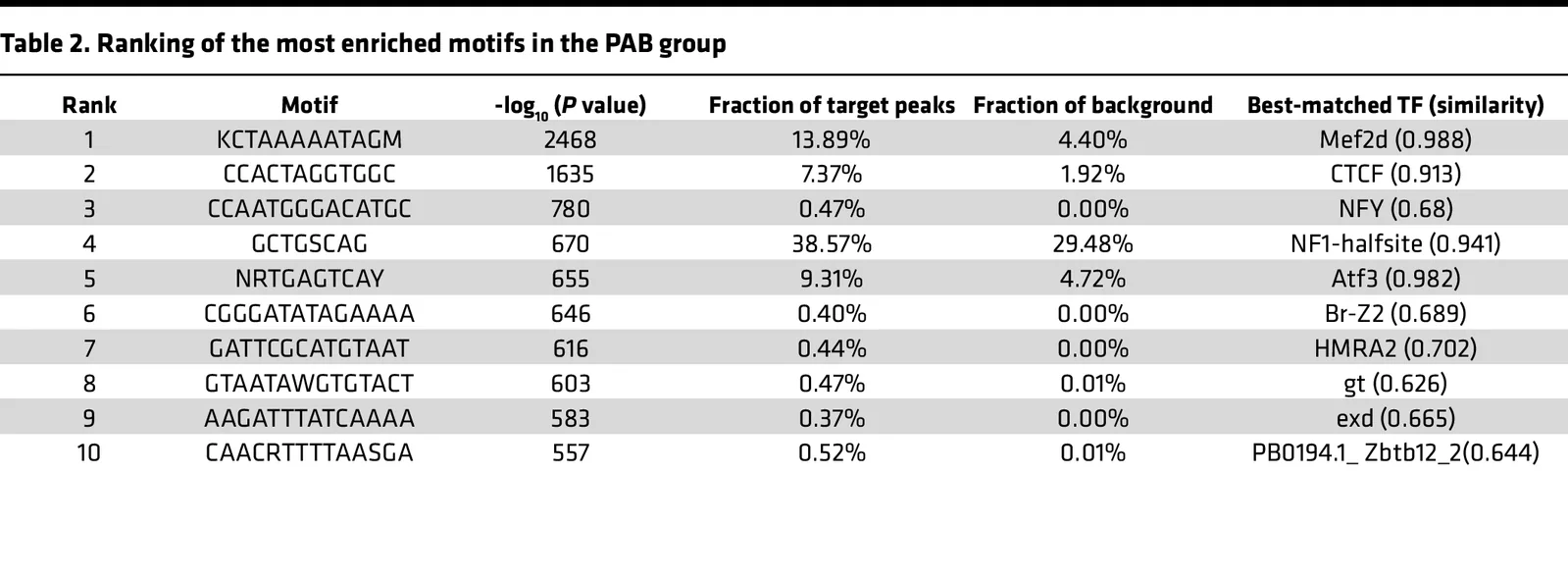
Ranking of the most enriched motifs in the PAB group

**Table 3:**
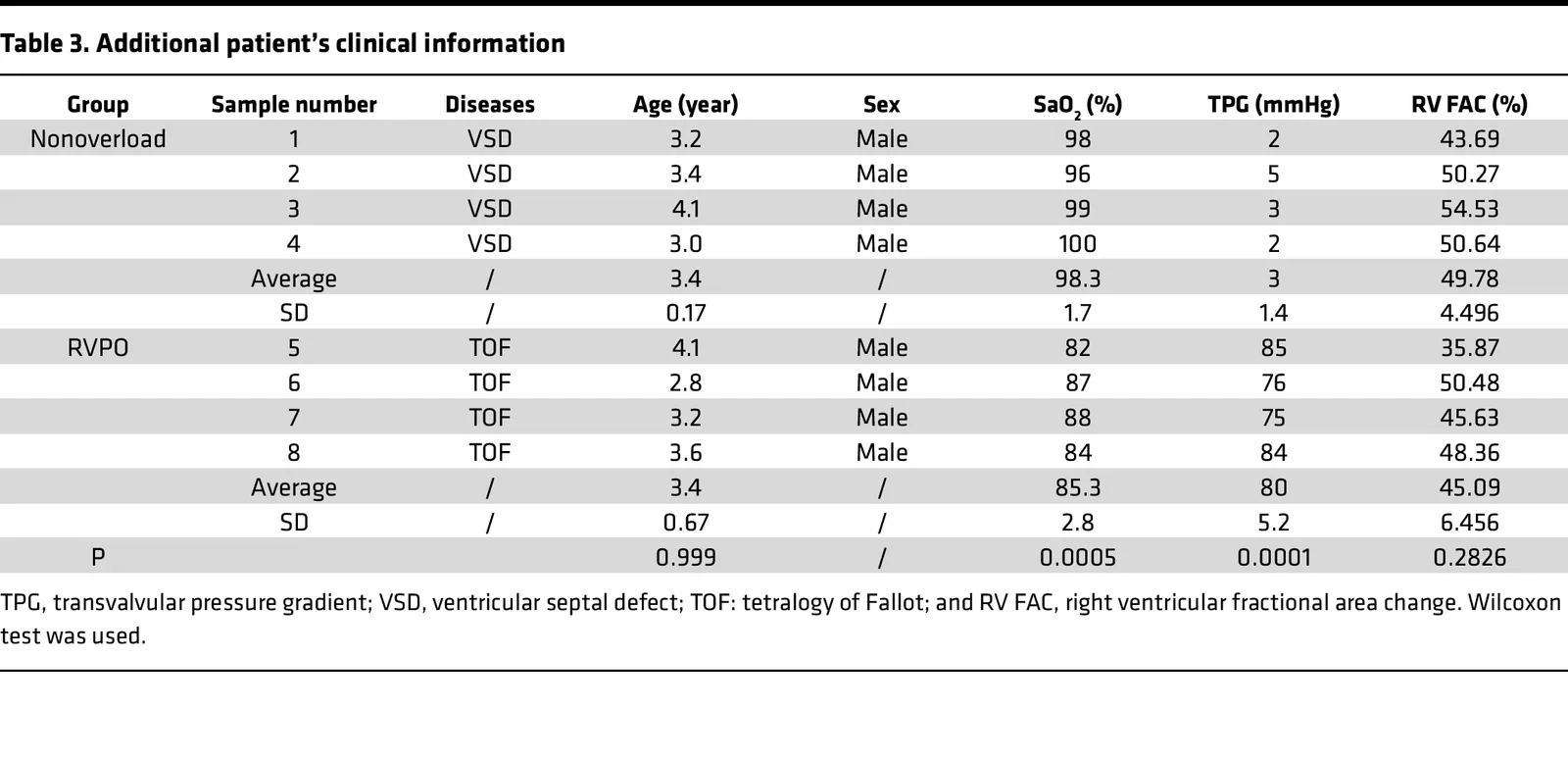
Additional patient’s clinical information

## Version 1 09/22/2026

In-Press Preview

## Version 2 09/22/2026

Electronic publication
