# Supplementary material for: Right ventricular pressure overload disturbs T-tubule maturation via MEF2D’s transcriptional regulation of BIN1
Source: JCI Insight. 2026 Sep 22;11(18):e204032. doi: 10.1172/jci.insight.204032 (PMC13596720; doi:10.1172/jci.insight.204032)
Supplement: Supplementary file 1 [file jciinsight-11-204032-s249.pdf]

Supplemental Figure 1. Establishment of the neonatal PAB model.

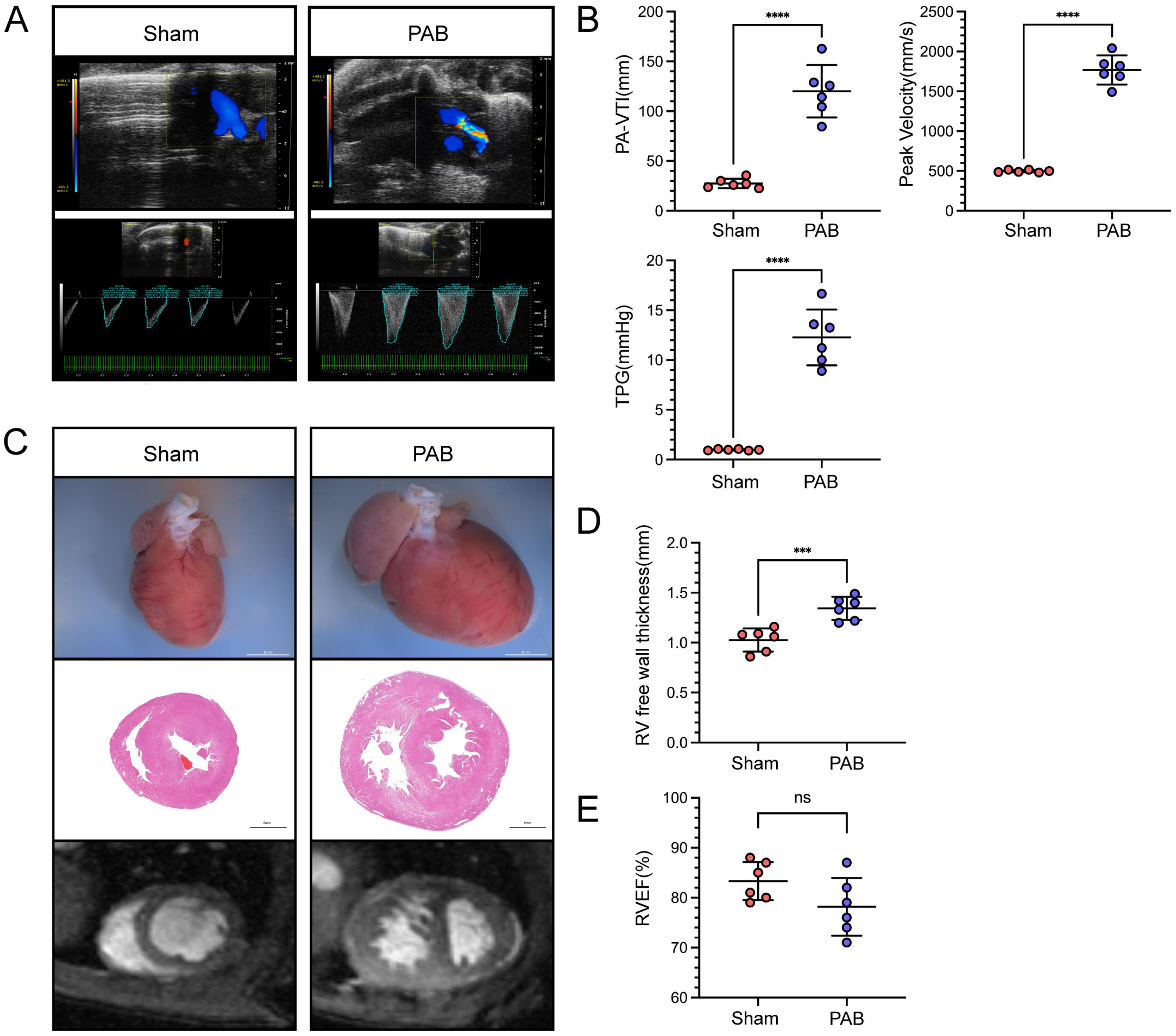

(**A**) Representative echocardiographic images at postnatal day 7(P7) after sham or PAB surgery in neonatal rats. (**B**) Quantification of pulmonary artery (PA) velocity time integral (VTI) , PA-peak velocity and transvalvar pressure gradient (TPG) between the sham and PAB groups. N=6 rats. (**C**) Representative gross images, H&E-stained two-chamber cross-sections and MRI images of the P21 heart in the Sham and PAB rats. Quantification of the thickness of the RV free wall (**D**)and RV ejection fraction (EF) (%) (E) in the sham and PAB groups. N=6 rats, Data are presented as mean ± SD. P values were calculated by 2-tailed Student's *t* test. Ns=no significance, ***P < 0.001, ****P < 0.0001.

Supplemental Figure 2. Single-cell analysis uncovers the relationship between hypertrophic marker genes and T-tubule genes.

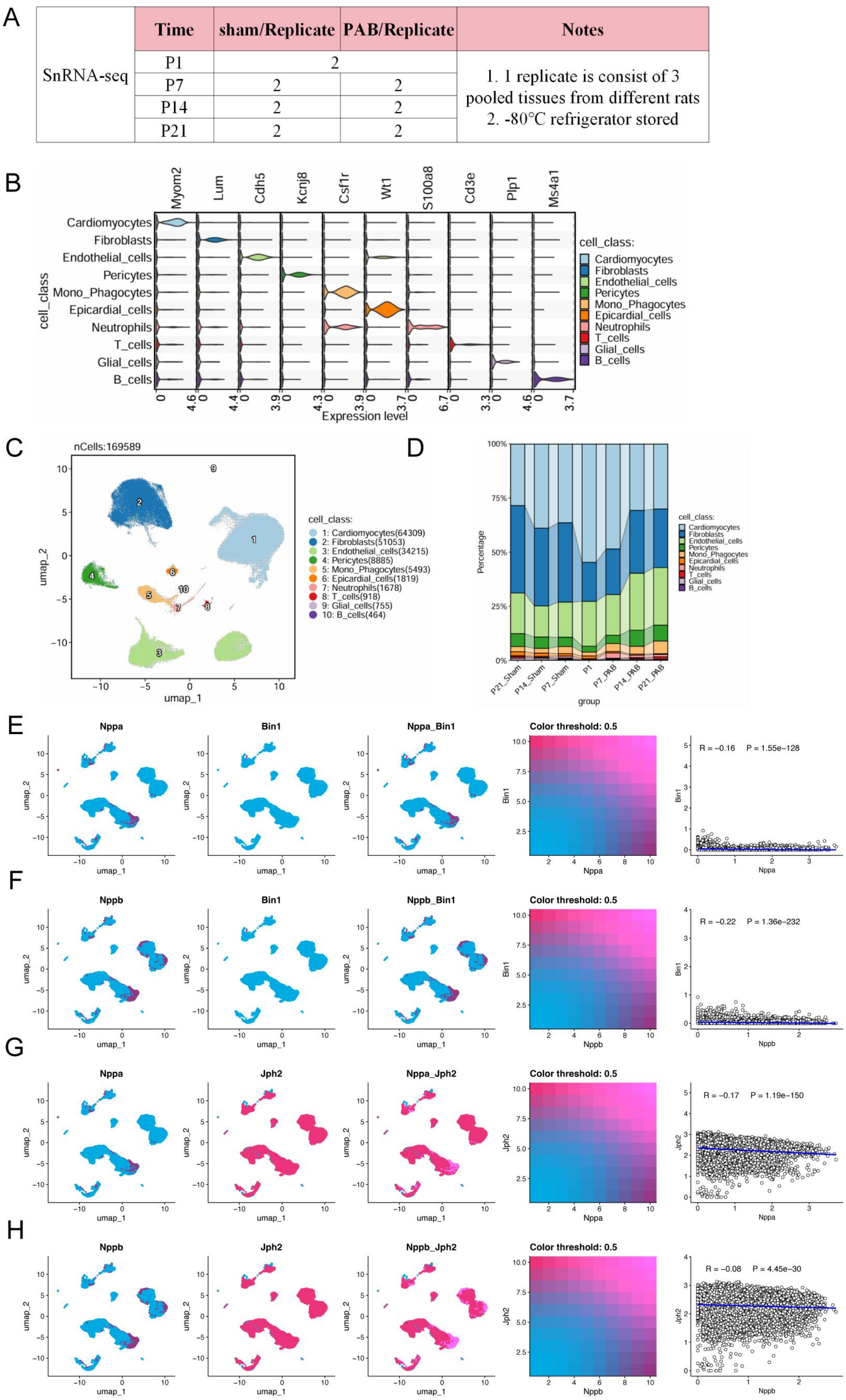

(**A**) Experimental design of single-nucleus RNA-sequencing. (**B**) Marker gene expression levels for each cell type. (**C**) UMAP analysis revealing 10 distinct cell types. (**D**) Temporal changes in the proportions of major cell types. At P7, the proportion of cardiomyocytes was significantly higher in the PAB group than in the sham group, but subsequently decreased, while the proportion of endothelial cells gradually increased. (**E**) Correlation analysis between *Bin1* and *Nppa* expression levels. (**F**) Correlation analysis between *Bin1* and *Nppb* expression levels. (**G**) Correlation analysis between *Jph2* and *Nppa* expression levels. (**H**) Correlation analysis between *Jph2* and *Nppb* expression levels.

Supplemental Figure 3. Expression and chromatin accessibility of *Jph2*.

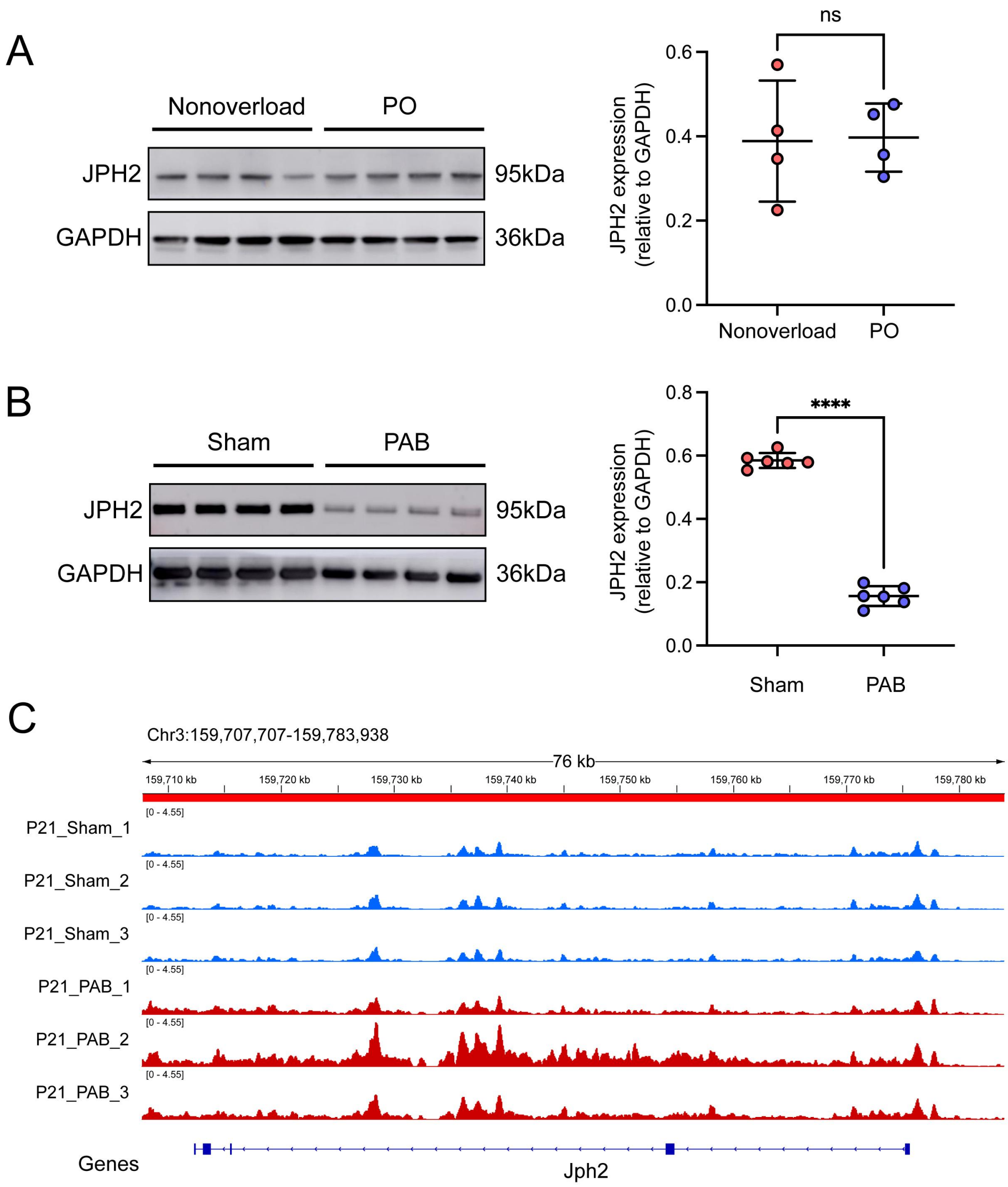

(**A**) Left panel: Representative JPH2 immunoblot in VSD and TOF patient samples. Right panel: Quantification of JPH2 expression in human samples (n = 4 patients per group; Data are presented as mean ± SD. P values were calculated by 2-tailed Student's *t* test). Ns=no significance, (**B**) Left panel: Representative image of JPH2 western blot in the sham and PAB groups. Right panel: Quantification of JPH2 protein expression in the sham and PAB groups. N=6 rats, Data are presented as mean ± SD. P values were

calculated by 2-tailed Student's *t* test. ****$P < 0.0001$. (**C**) Visual analysis of chromatin accessibility in adjacent regions of the transcription start site (TSS) of the *Jph2* gene.

Supplemental Figure 4. Cardiomyocyte-specific BIN1 overexpression and MEF2D knockdown reduce the cross-sectional area of cardiomyocytes.

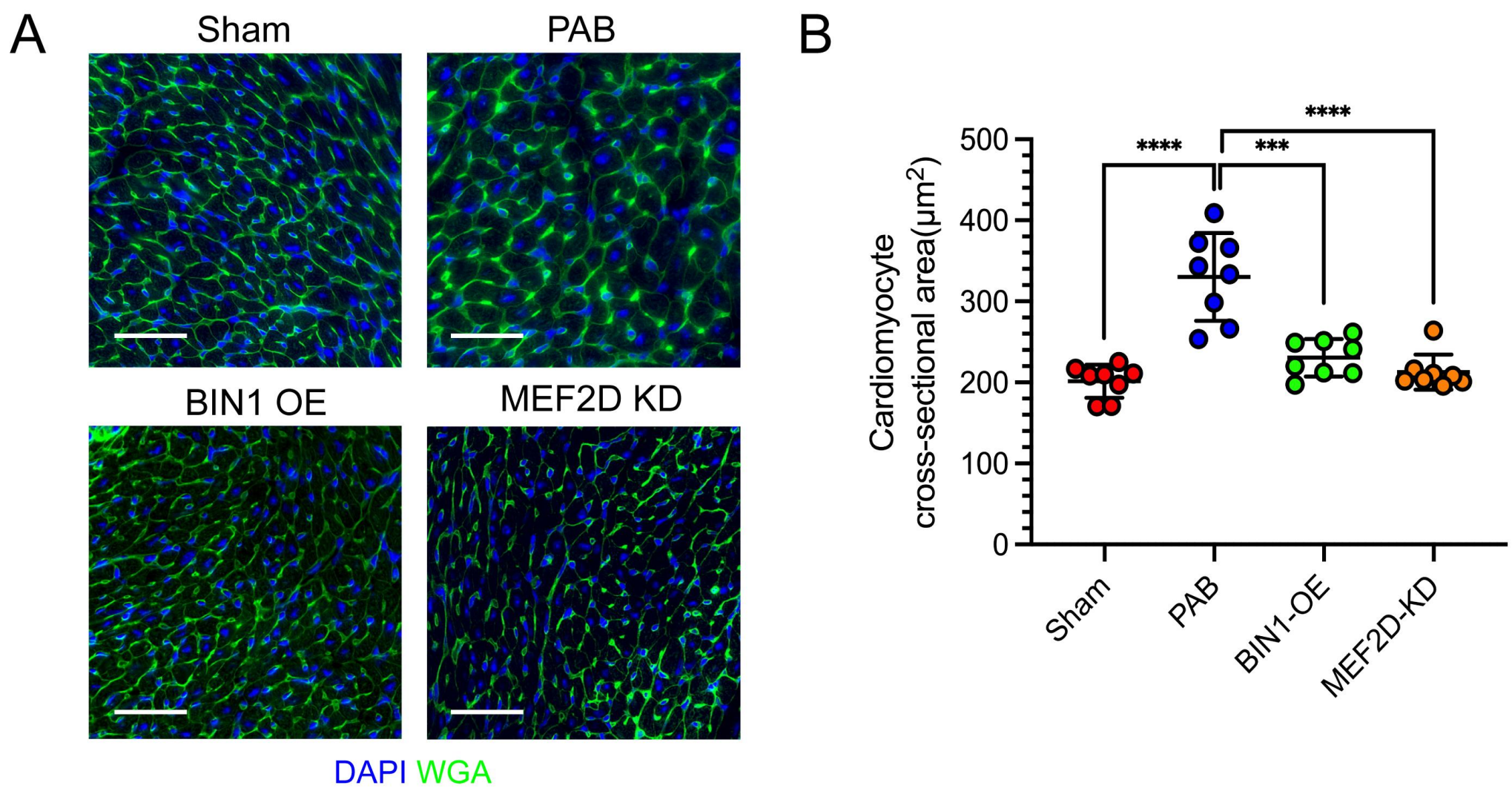

(**A**) Representative image of WGA staining of cardiomyocytes in sham, PAB, Bin-OE and MEF2D-KD groups. Scale bar: 50μm. (**B**) Quantification of the cardiomyocyte cross-sectional area of in the sham, PAB, BIN1-OE and MEF2D-KD groups. For each rat, 25 randomly selected cardiomyocytes were measured, and the mean value was used as one biological replicate (n = 8 rats per group). Data are presented as mean ± SD. P values were calculated by 1-way ANOVA with post hoc Holm-Šídák test. ***$P < 0.001$, ****$P < 0.0001$.

Supplemental Figure 5. **MEF2D expression levels are significantly increased in human TOF samples.**

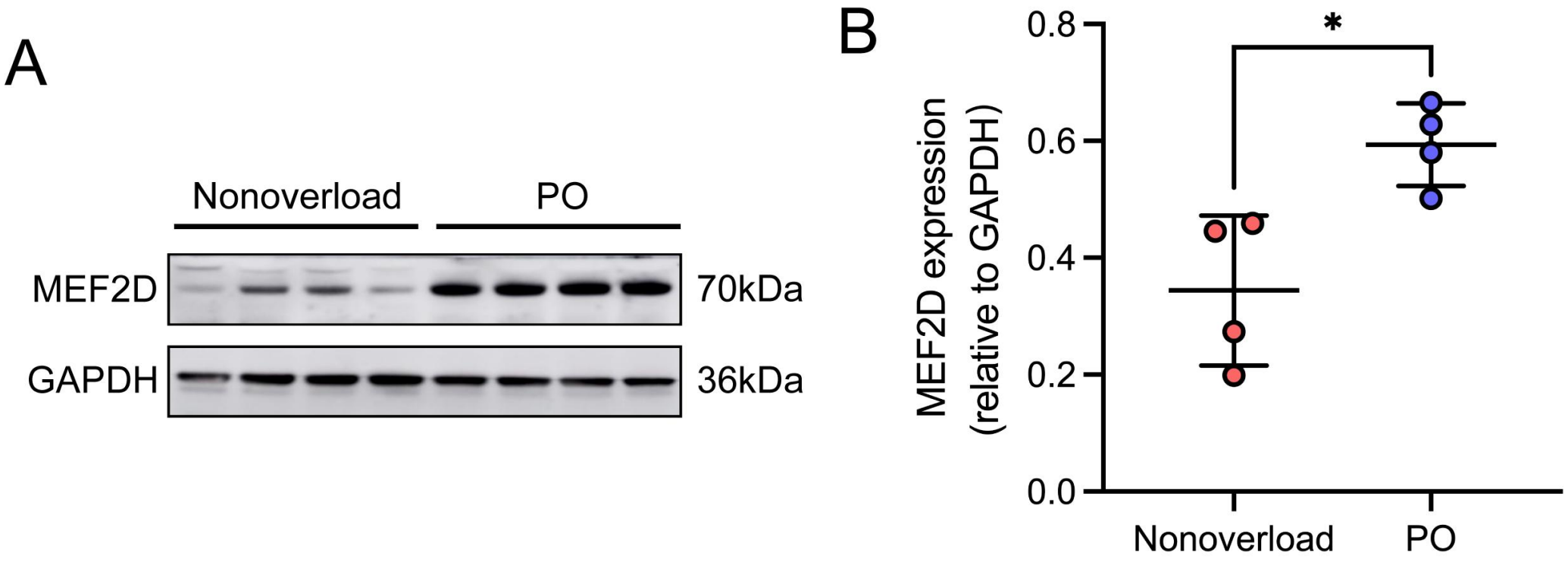

(**A**) Representative MEF2D immunoblot in VSD and TOF patient samples. (**B**) Quantification of MEF2D expression in human samples (n = 4 patients per group; Data are presented as mean ± SD. P values were calculated by 2-tailed Student's *t* test). $*P < 0.05$

Supplemental Figure 6. Structural differences between pBIN1 and cBIN1 isoforms.

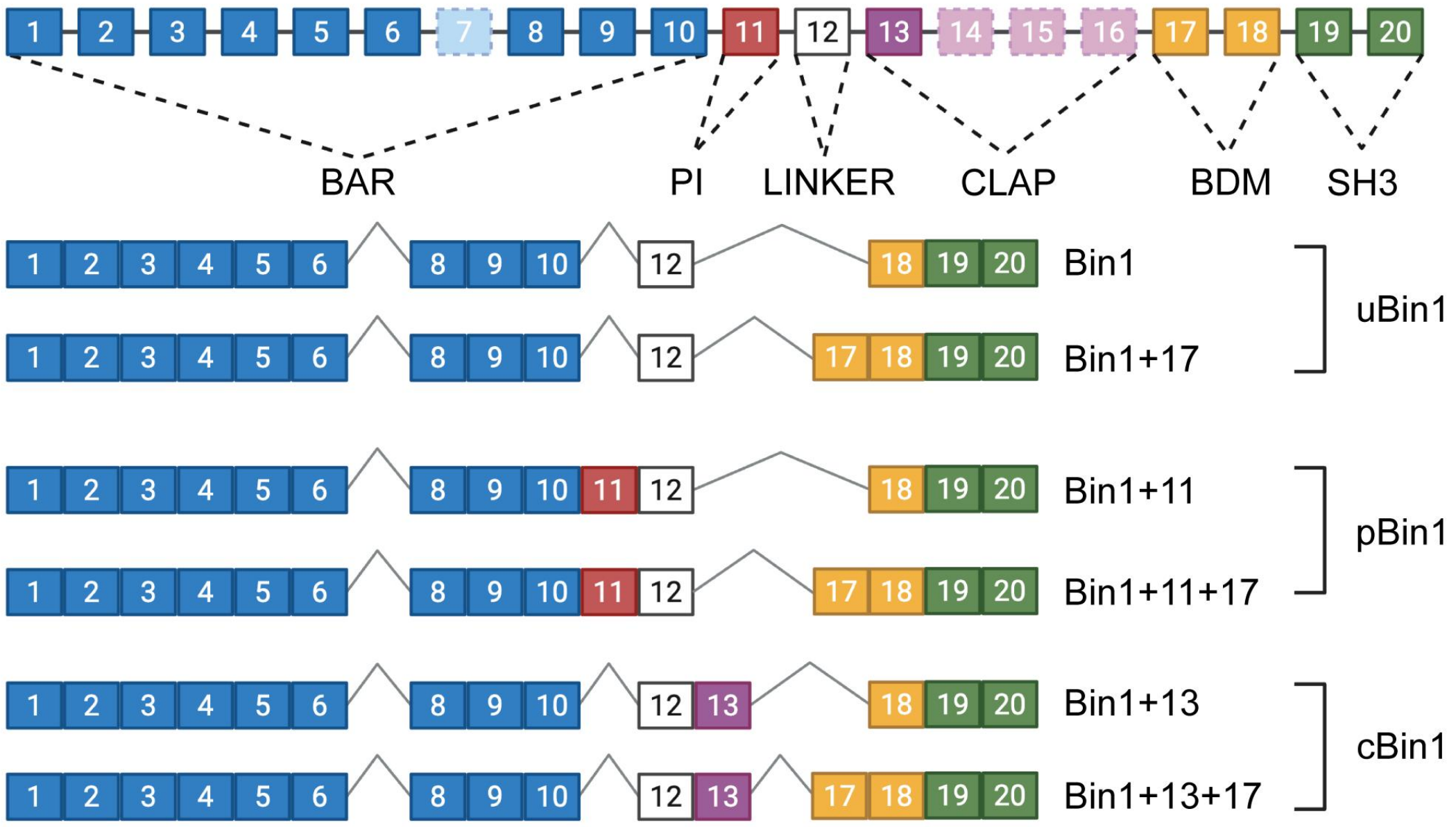

Schematic illustration of *Bin1* mRNA splicing isoforms identified in cardiomyocytes. N, N-terminal amphipathic helix domain; BAR, BIN1-Amphiphysin-Rvs167 domain; PI, phosphoinositide-binding domain; CLAP, clathrin-associated protein-binding domain; MBD, Myc-binding domain; SH3, Src homology 3 domain.
