# Supplementary material for: Right ventricular pressure overload disturbs T-tubule maturation via MEF2D’s transcriptional regulation of BIN1
Source: JCI Insight. 2026 Sep 22;11(18):e204032. doi: 10.1172/jci.insight.204032 (PMC13596720; doi:10.1172/jci.insight.204032)

Figure 3E

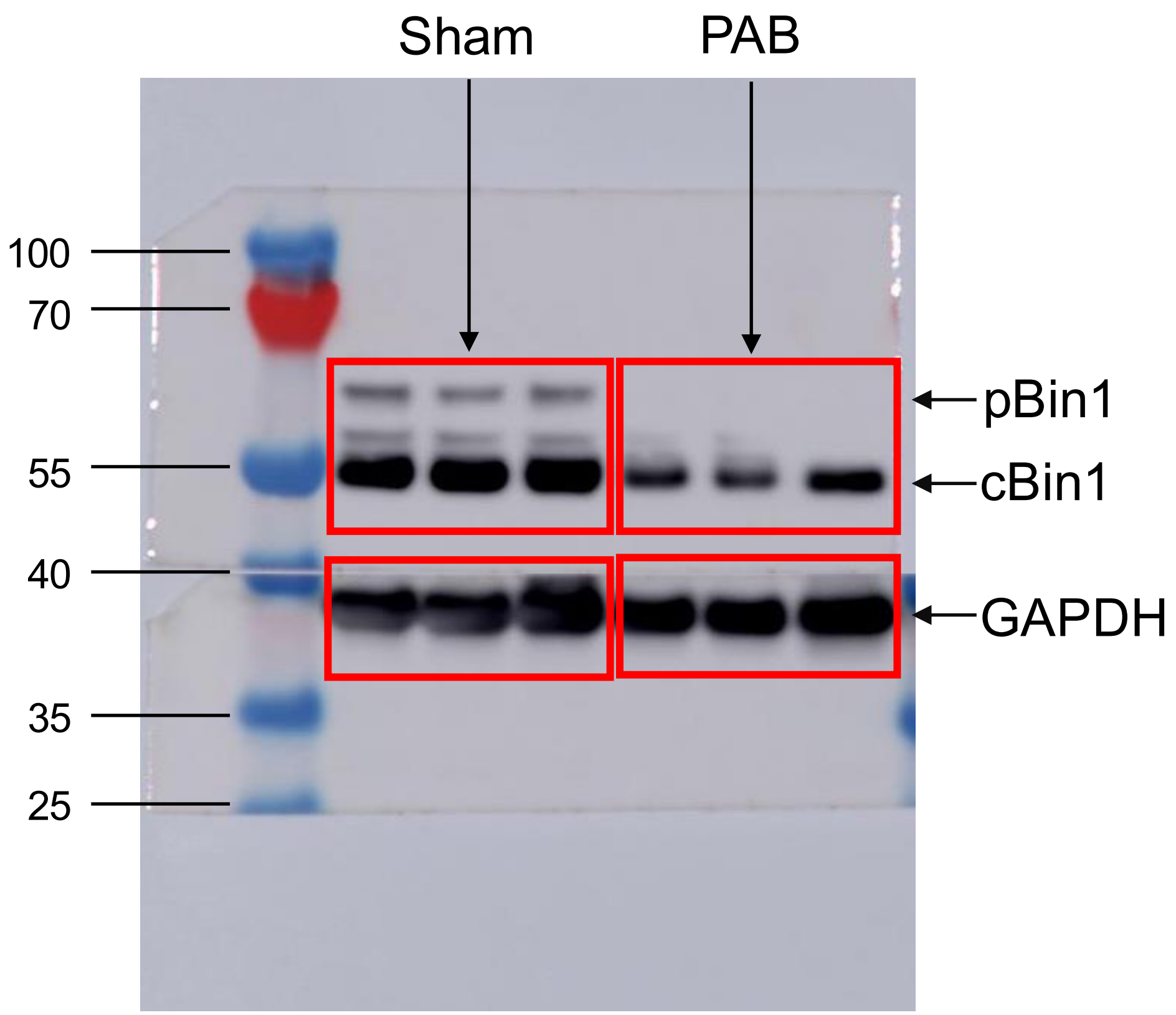

Figure 3H

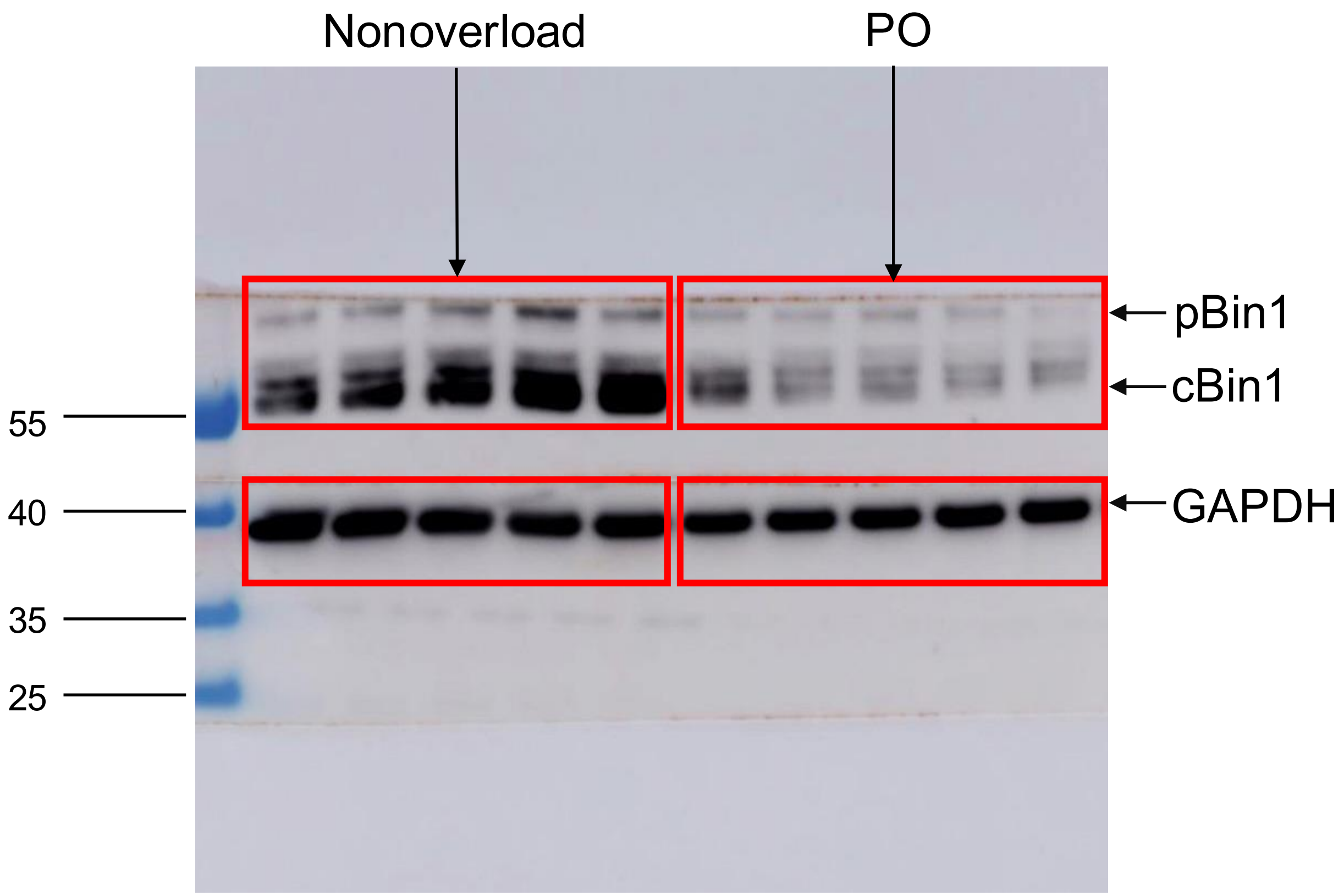

Figure 4D

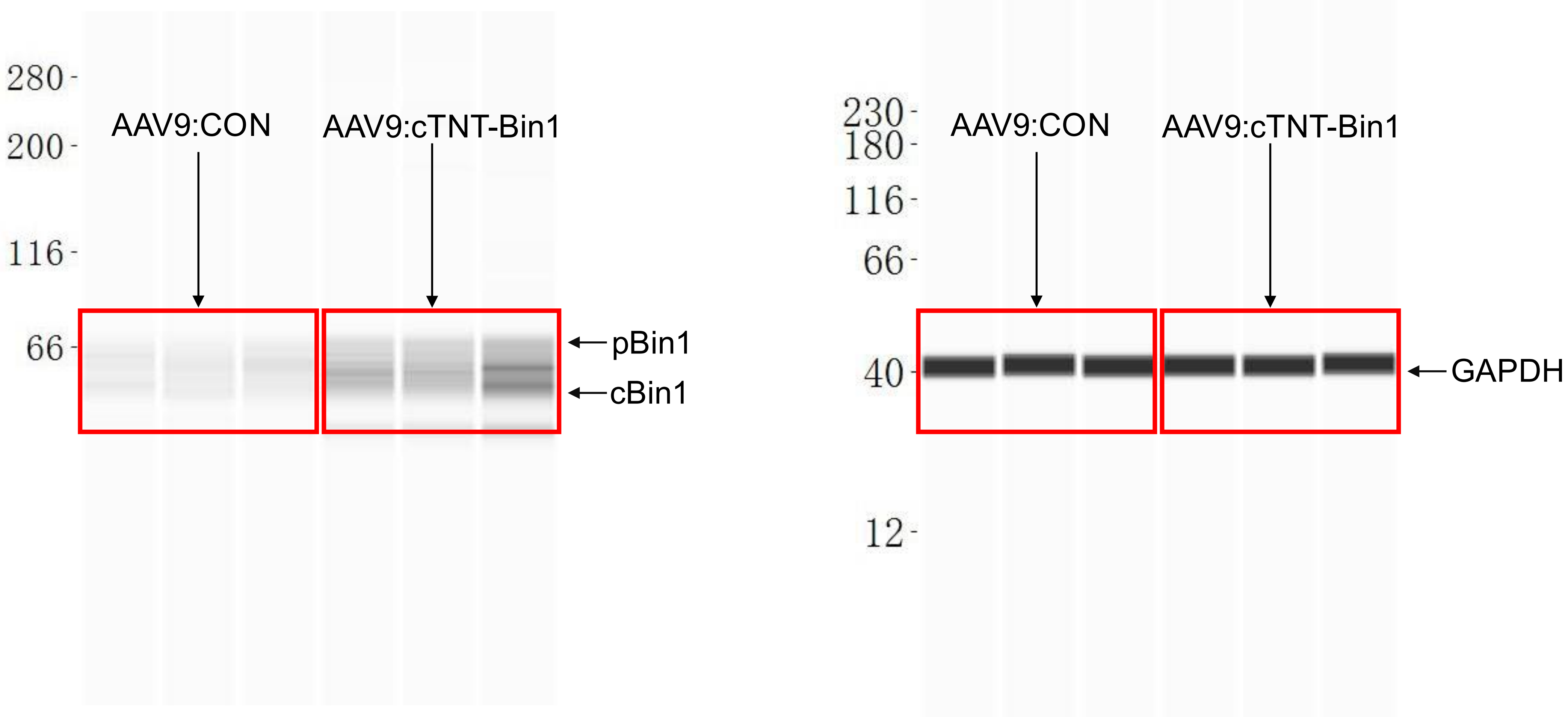
280
200
116
66
AAV9:CON
AAV9:cTNT-Bin1
pBin1
cBin1
230
180
116
66
40
12
AAV9:CON
AAV9:cTNT-Bin1
GAPDH

Figure 6D

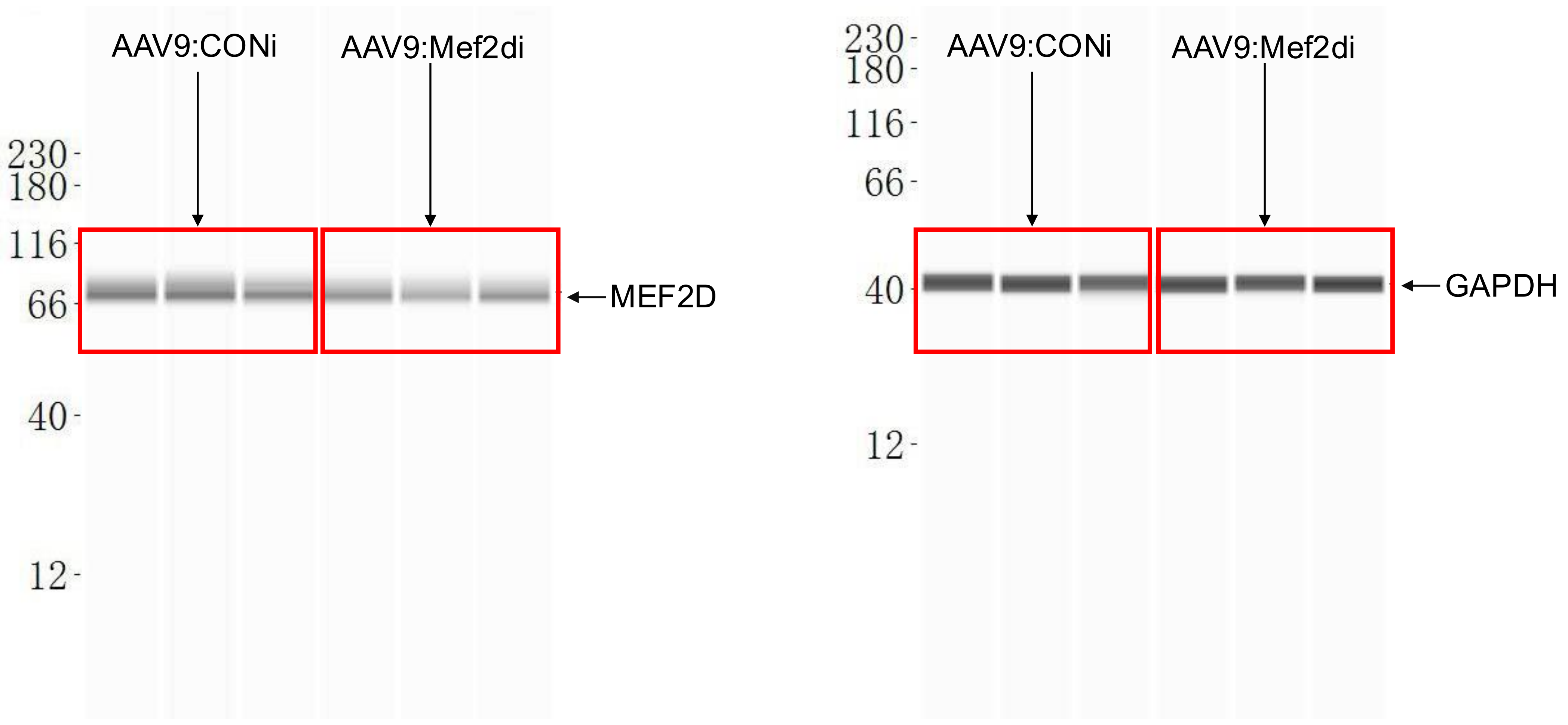
AAV9:CONi
AAV9:Mef2di
230
180
116
66
40
12
MEF2D
AAV9:CONi
AAV9:Mef2di
230
180
116
66
40
12
GAPDH

Figure 6H

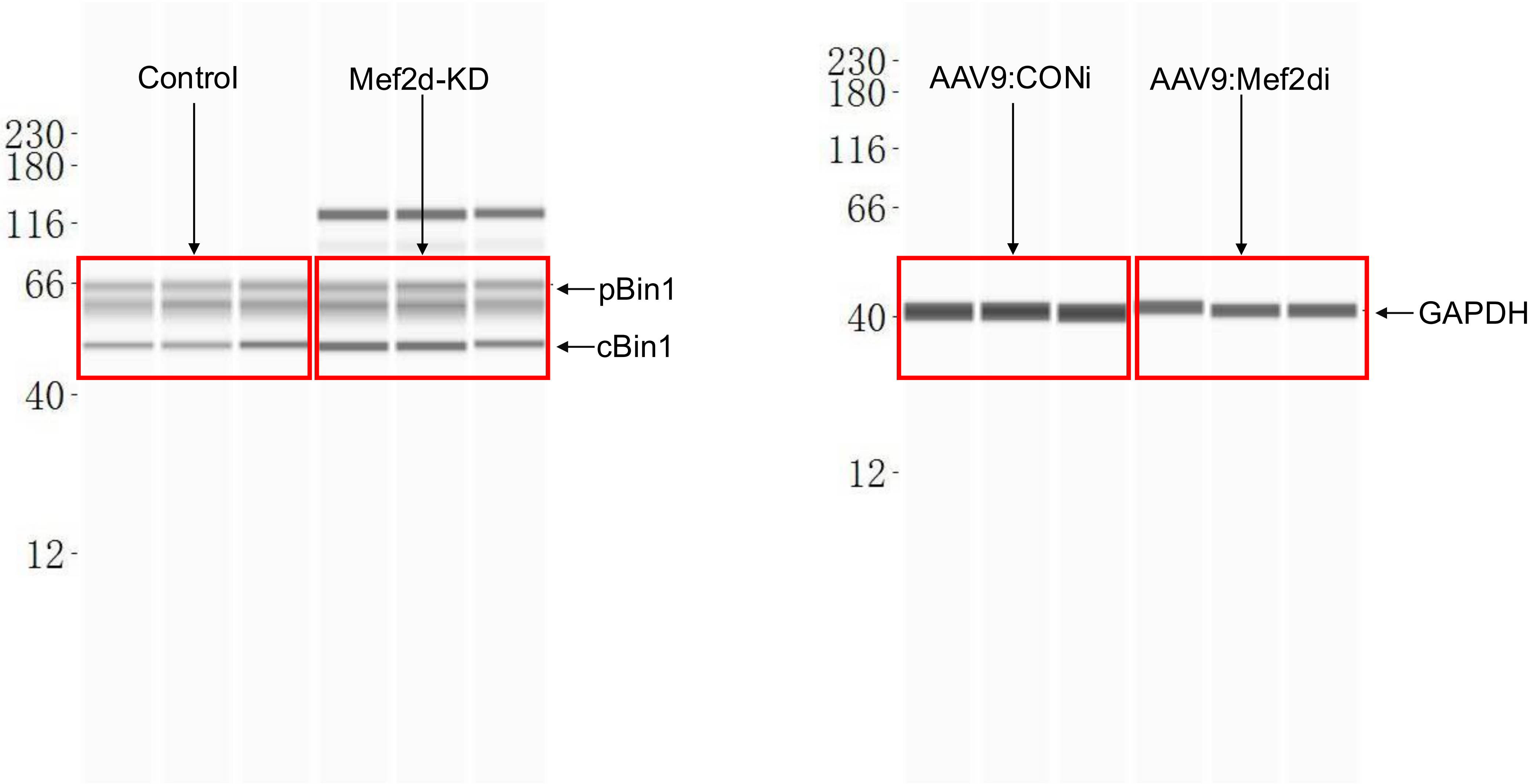

Control
Mef2d-KD
230
180
116
66
40
12
pBin1
cBin1
AAV9:CONi
AAV9:Mef2di
230
180
116
66
40
12
GAPDH

Supplemental Figure 3A

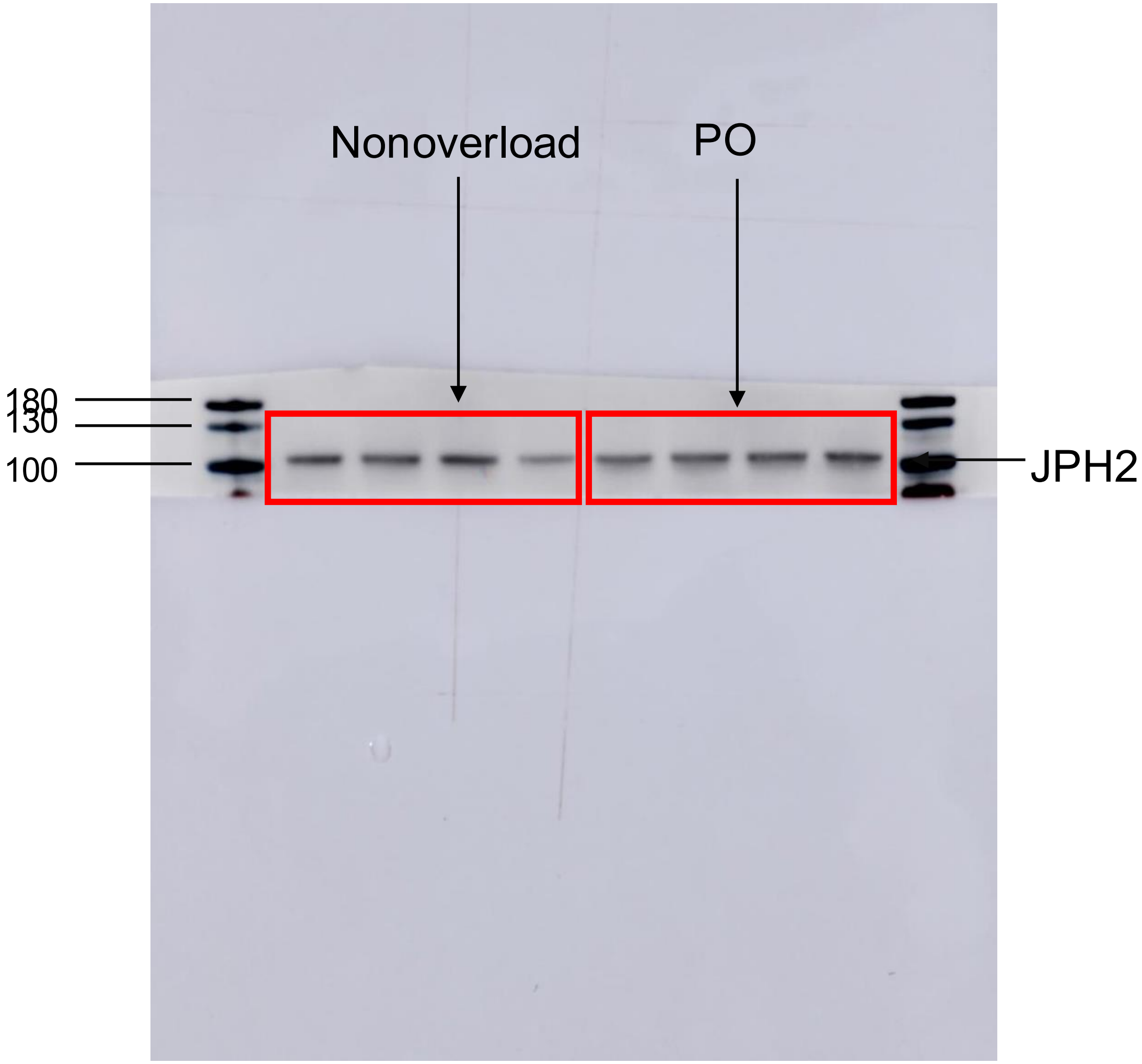

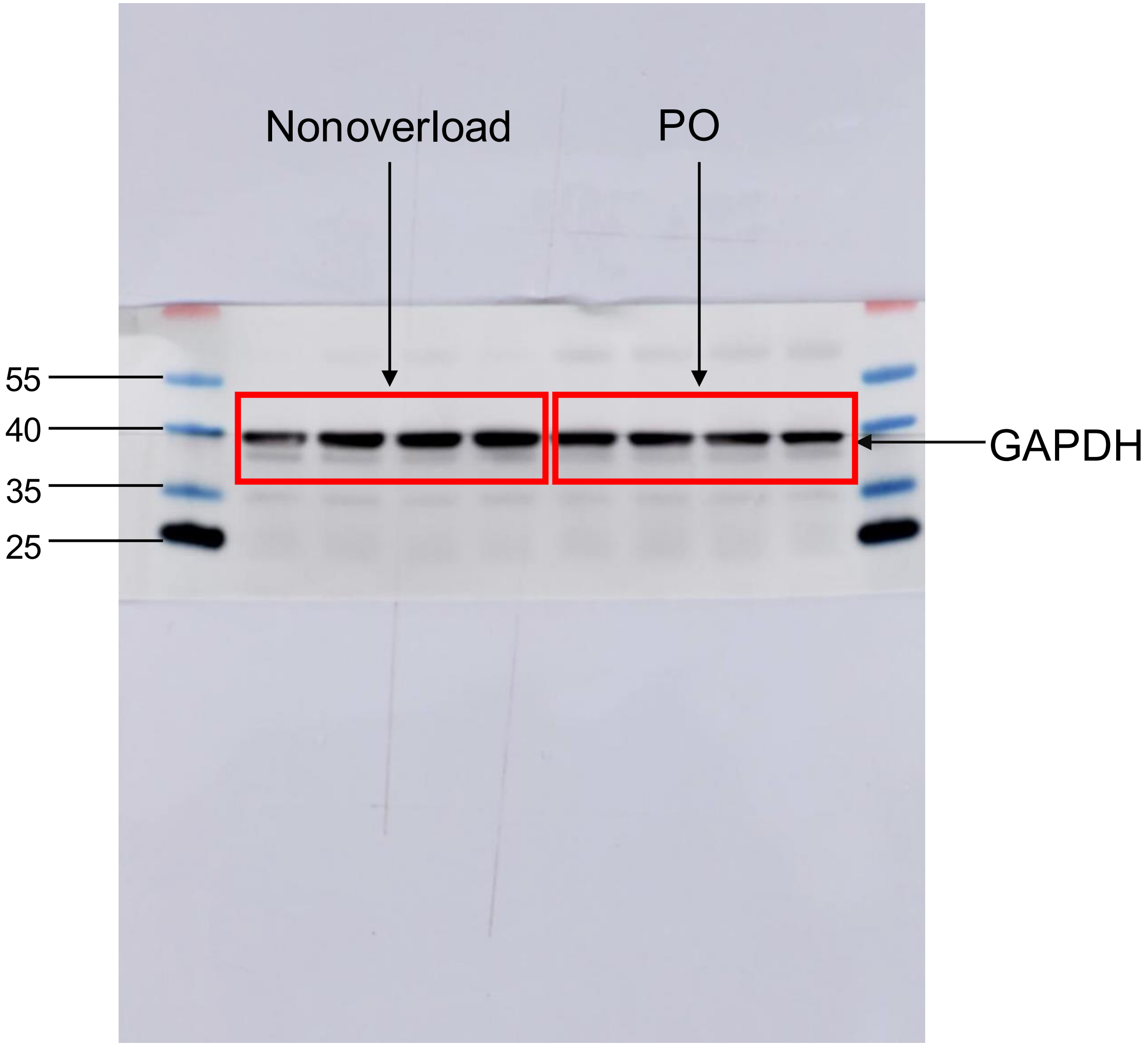

Supplemental Figure 3B

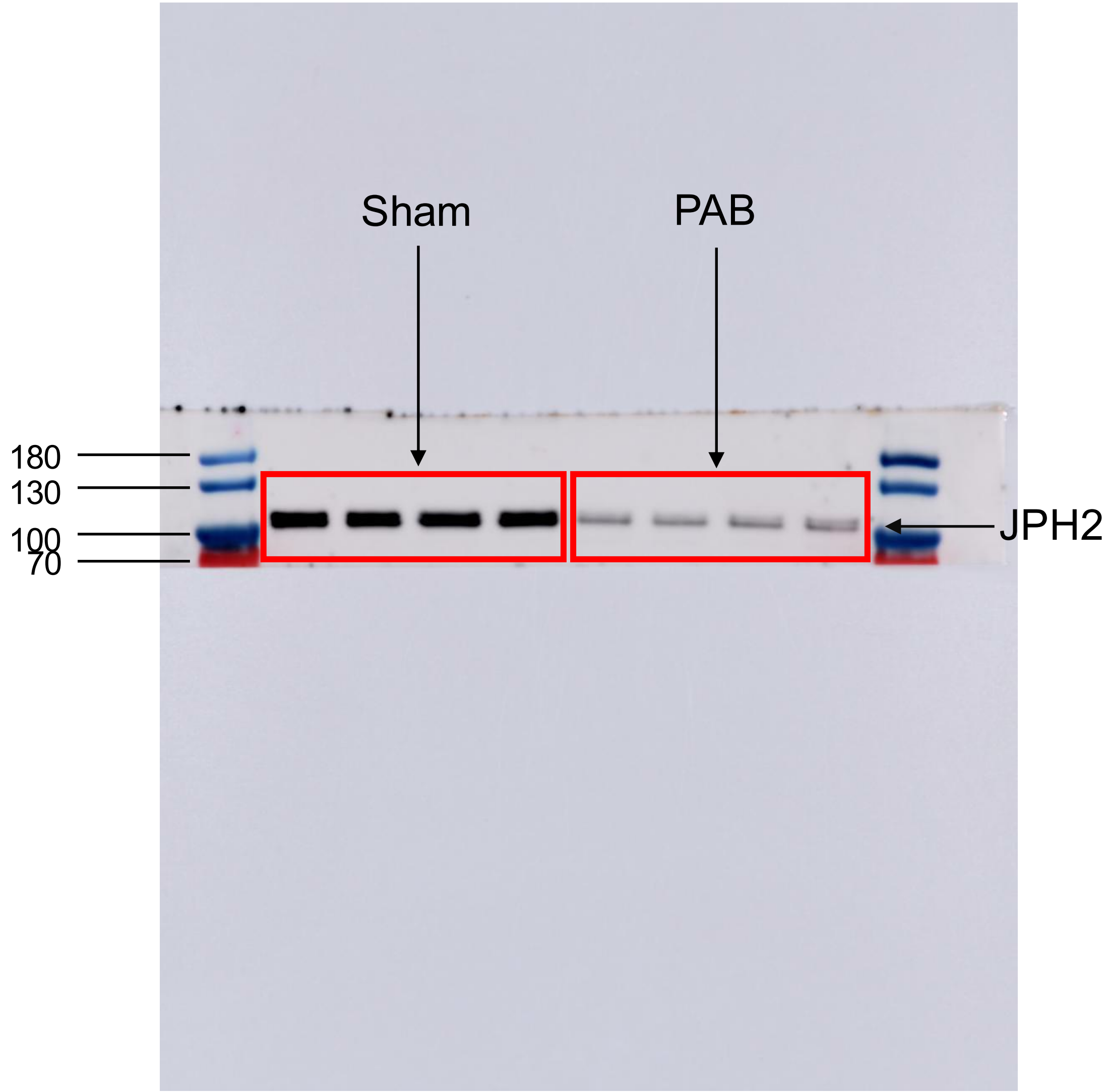

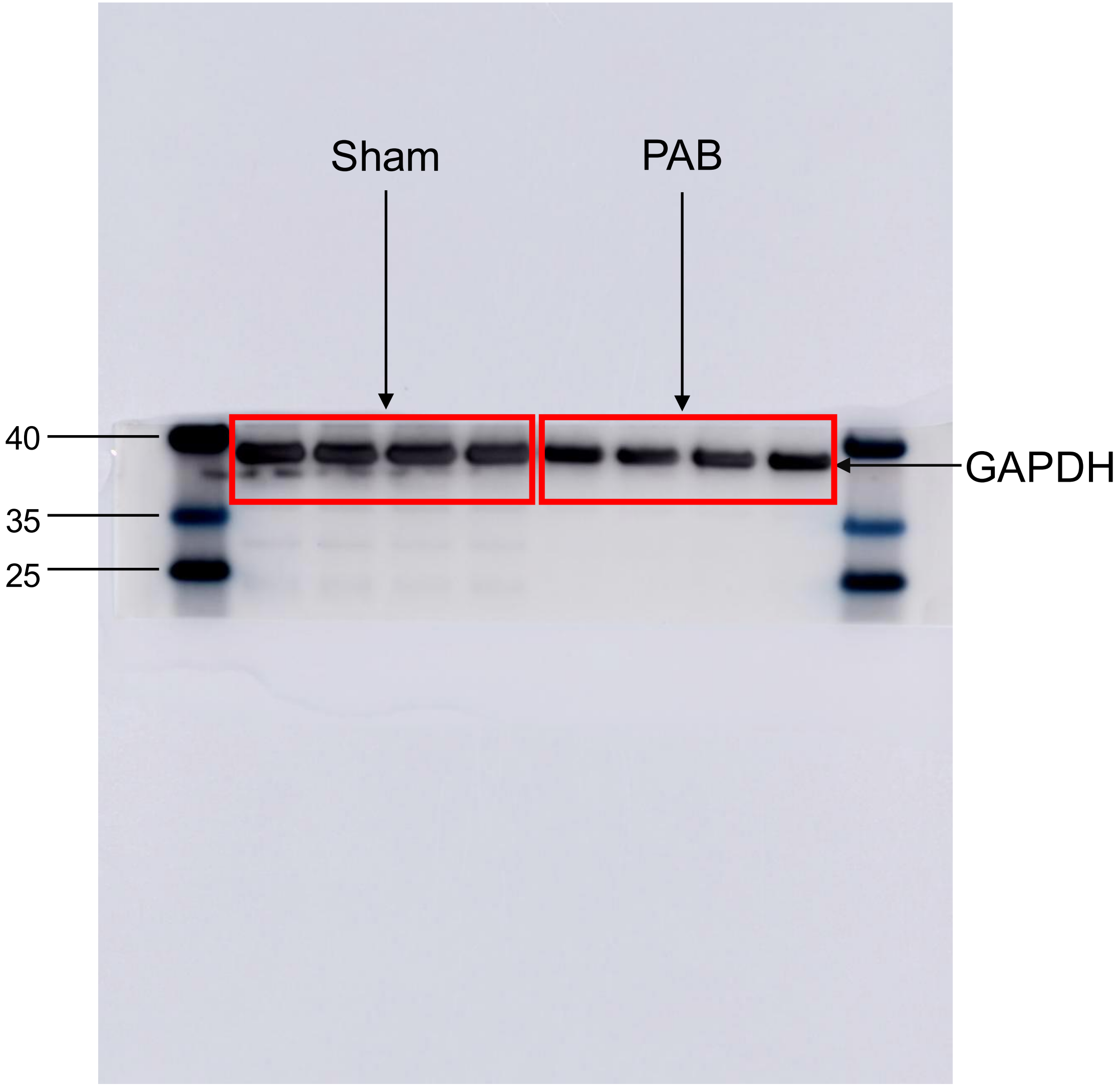

Supplemental Figure 5A

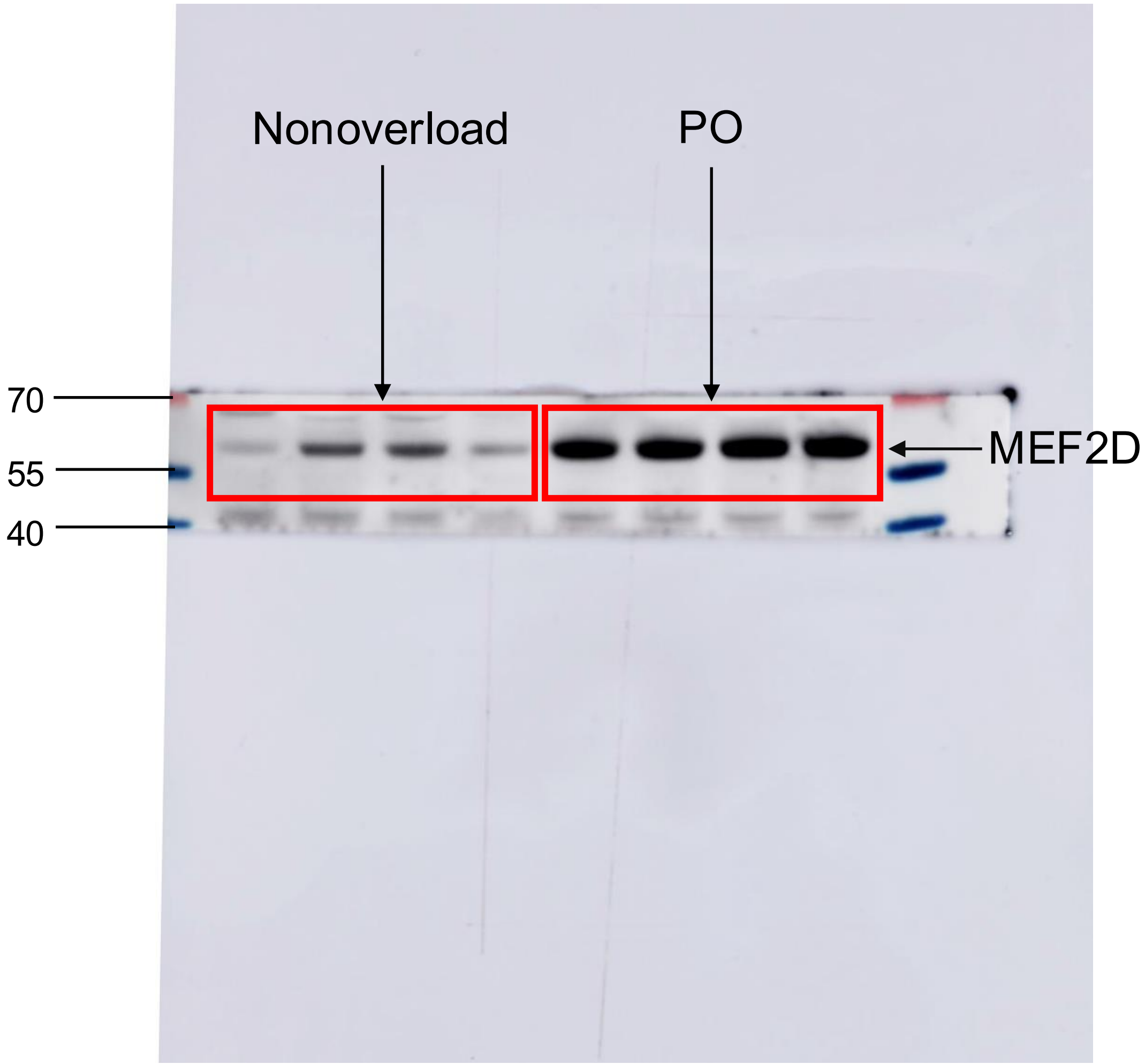

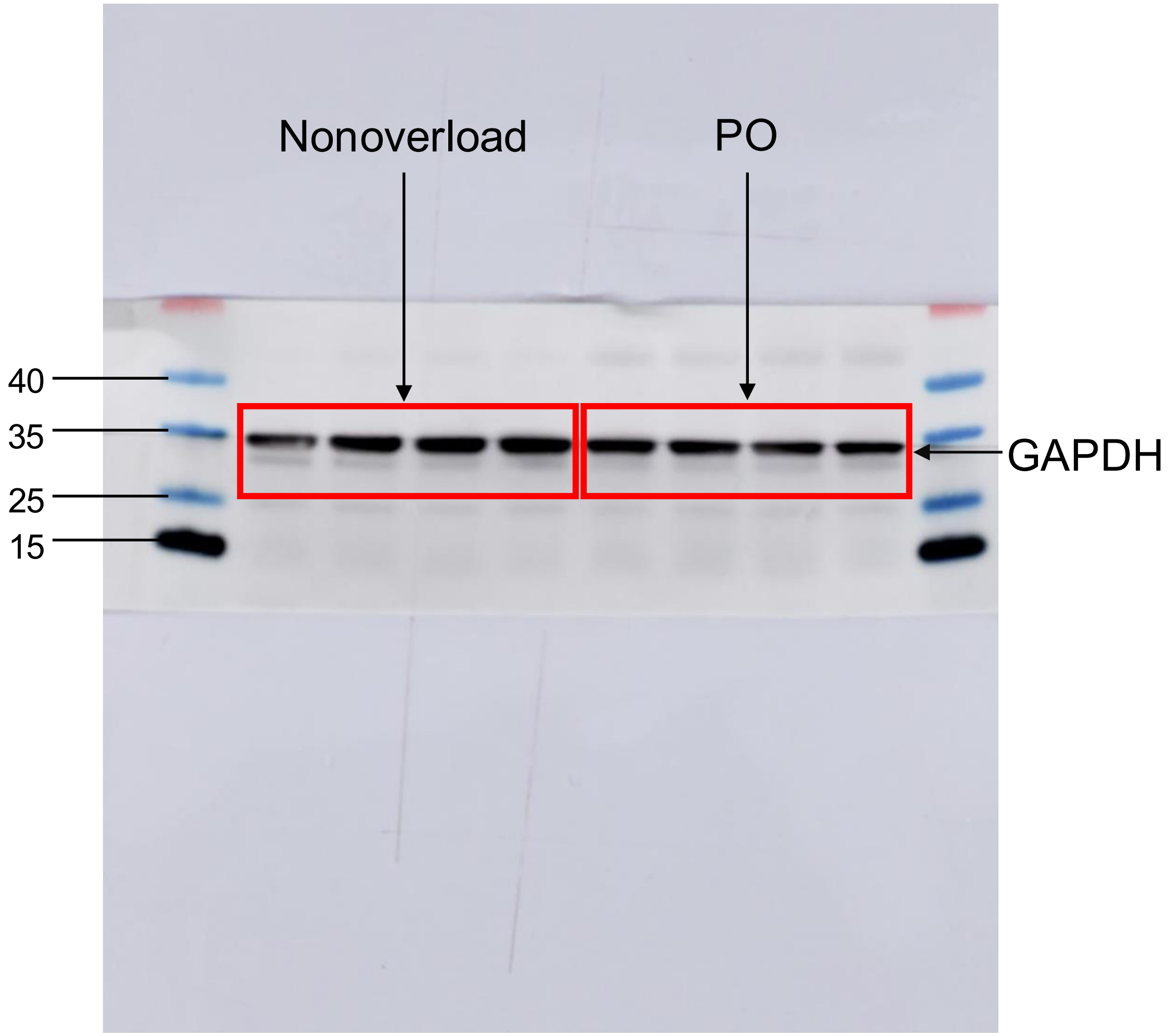

Supplement: Supplementary file 2 [file jciinsight-11-204032-s250.pdf]
